# Supplementary material for: Symmetry-breaking host–guest assembly in a hydrogen-bonded supramolecular system
Source: Nat Commun. 2023 Jan 11;14:155. doi: 10.1038/s41467-023-35850-4 (PMC9834293; doi:10.1038/s41467-023-35850-4)
Supplement: Supplementary file 1 — Supplementary Information [file 41467_2023_35850_MOESM1_ESM.pdf]

Supplementary Information

Symmetry-Breaking Host–guest Assembly in a Hydrogen-bonded Supramolecular System

Shinnosuke Horiuchi*[,1,2,3], Takumi Yamaguchi[4], Jacopo Tessarolo[2], Hirotaka Tanaka[1], Eri Sakuda[1,2], Yasuhiro Arikawa[1], Eric Meggers[5], Guido H. Clever*[,2], Keisuke Umakoshi*[,1]

[1]Division of Chemistry and Materials Science, Graduate School of Engineering, Nagasaki University, 1-14 Bunkyo-machi, Nagasaki 852-8521, Japan.

[2]Department of Chemistry and Chemical Biology, TU Dortmund University, Otto-Hahn-Straße 6, 44227 Dortmund, Germany.

[3]Department of Basic Science, Graduate School of Arts and Sciences, The University of Tokyo, 3-8-1 Komaba, Meguro-ku, Tokyo 153–8902, Japan.

[4]School of Materials Science, Japan Advanced Institute of Science and Technology, 1-1 Asahidai, Nomi 923–1292, Japan.

[5]Fachbereich Chemie, Philipps-Universität Marburg, Hans-Meerwein-Straße 4, 35043 Marburg, Germany.

*E-mail: shoriuchi@g.ecc.u-tokyo.ac.jp, kumks@nagasaki-u.ac.jp

Table of Contents

- **Materials** 2
- **Synthetic procedure of the components** 2
- **General procedure of encapsulation experiments** 4

Plausible self-assembly and encapsulation mechanism of (**1a**)$_6$⊃([**2a**]Cl) 4

NMR and ESI-MS spectra of (**1**)$_6$⊃([**2a**]Cl) 5

A molecular model of (**1**)$_6$⊃[**2a**]$^+$ 12

NMR and ESI-MS spectra of (**1**)$_6$⊃([**2a**]NO_3) 13

NMR spectra of a mixture of (**1a**)$_6$ and [**2a**]PF_6 16

NMR and ESI-MS spectra of (**1**)$_6$⊃([**2b**]Cl) 16

NMR and ESI-MS spectra of a mixture of (**1**)$_6$ and [**2c**]Cl 18

^{1}H NMR and ESI-MS spectra of (**1**)$_6$⊃([**3a**]Cl_2) 20

^{1}H NMR and ESI-MS spectra of (**1**)$_6$⊃([**3b**]Cl_2). 22

Ion mobility spectra of [(**1b**)$_5$(**3a**)]$^{2+}$ and [(**1b**)$_5$(**3b**)]$^{2+}$ 23

- **X-ray Structural Determination** 24

Single crystal X-ray structure of (**1c**)$_3$⊃([**3a**]Cl_2) 26

- **Photophysical studies in the non-chiral system**

Photophysical data of [**2a**]Cl in the presence of **1a** 29

Photophysical data of [**2a**]NO_3 in the presence of **1a** 30

Photophysical data of [**2a**]PF_6 in the presence of **1a** 31

Photophysical data of [**2b**]Cl in the presence of 1a 32

Photophysical data of [**2c**]Cl in the presence of **1a** 33

Photophysical data of [**3a**]Cl_2 in the presence of **1a** 34

Photophysical data of [**3b**]Cl_2 in the presence of **1a** 35

Proposed photo-excited dynamics after host–guest formation 36

- **Photophysical studies in the chiral system**

^{1}H NMR spectra of host–guest complexes of Λ- and Δ-[**2a**]Cl 37

CD spectra of Λ/Δ-[**2a**]X (X = Cl, NO_3, PF_6) 37

UV-Vis, CD, and CPL data of Λ/Δ-[**2a**]Cl in the presence of **1a** 38

CD and CPL data of Λ/Δ-[**2a**]NO_3 in the presence of **1a** 39

CD and CPL data of Λ/Δ-[**2a**]PF_6 in the presence of **1a** 39

CD and CPL data of Λ/Δ-[**2b**]Cl in the presence of **1a** 40

Frontier orbitals of a Ir complex cation 41

Proposed mechanism of the symmetry-breaking effect 41

- **Supplementary References** 42

Materials

The components, resorcin[4]arene **1a** and **1c**, racemic and non-racemic coordination complexes were prepared according to literature methods and purified by recrystallization before usage.[1–4] Other commercially available compounds were used as received without further purification.

Synthetic procedure of the components

Synthesis of resorcin[4]arene 1a. Resorcin[4]arene **1a** was synthesized according to the previously reported procedure.[1] Resorcinol (2.00 g, 18.2 mmol) was dissolved in EtOH (12 mL) and 3.6 mL of conc.-HCl (37%) was added to the solution. The solution was carefully degassed with N_2 bubbling for 5 min. EtOH solution (10 mL) of dodecanal (3.35 g, 18.2 mmol) was added dropwise to the solution over a 20-min period with stirring at 0 ºC under N_2 atmosphere. The colorless solution was allowed to warm to room temperature. The solution was gradually heated and stirred at 80 ºC for 14 h under N_2 atmosphere. During the reaction, the solution turned red and some precipitate appeared. After the reaction completed, the reaction mixture was cooled to room temperature and pored into H_2O (50 mL) to give yellow precipitate. The solid was collected by filtration, washed with cold 50% EtOH-H_2O solution and dried in vacuo. Crude product **1a** was obtained as a pale yellow solid (3.87 g), which was purified by recrystallization from acetone-H_2O solution. Resorcin[4]arene **1a** was isolated as a colorless platelet crystals (40% isolated yield).

Synthesis of resorcin[4]arene 1c. Resorcin[4]arene **1c** was obtained in a similar procedure of **1a** with isovaleraldehyde instead of dodecanal. Resorcin[4]arene **1c** was purified by recrystallization from Et_2O solution of the crude **1c** and isolated as a colorless platelet crystals (32% isolated yield).

Synthesis of racemic Ir complex salts [2a]X (X = Cl, NO_3, and PF_6). Racemic Ir complex salts [**2**]Cl were synthesized according to a previously reported procedure.[2] $IrCl_3·3H_2O$ (150 mg, 0.06 mmol) and 2-phenylpyridine (150 mg, 0.12 mmol) were combined in a mixture of 2-ethoxyethanol (10 mL) and water (10 mL). The mixture was stirred at 120 ºC for 24 h under N_2 atmosphere to provide a yellow solution. After the solution was cooled to room temperature, the precipitate was collected by filtration and washed with water and hexane. The dinuclear bis-cyclometalated Ir(III) precursor complex, $[Ir_2(ppy)_4(\mu\text{-}Cl)_2]$, was obtained in a yield of 150 mg. The dinuclear Ir complex (107 mg, 0.10 mmol) and 4,-4'-di-*tert*-butyl-2,2'-bipyridine (59.1 mg, 0.22 mmol) were dissolved in a mixture of CH_2Cl_2 (10 mL) and MeOH (10 mL). After the mixture was heated at 50 ºC for 5 h under N_2, yellow solid was obtained by addition of Et_2O into the solution. Racemic Ir complex salt [**2a**]Cl was purified by recrystallization from CH_2Cl_2-hexane solution (85% isolated yield). The NO_3 and PF_6 salts were obtained by the reaction of the Cl salt with $AgNO_3$ and $AgPF_6$, respectively.

Synthesis of racemic Ir complex salt [2b]Cl. Racemic Ir complex salt [**2b**]Cl was obtained in a similar procedure as described for [**2a**]Cl with 4,-4'-dimethyl-2,2'-bipyridine instead of 4,-4'-di-*tert*-butyl-2,2'-bipyridine. Racemic Ir complex salt [**2b**]Cl was purified by recrystallization from CH_2Cl_2-hexane solution (90% isolated yield).

Synthesis of racemic Ir complex salt [2c]Cl. Racemic Ir complex salt [**2c**]Cl was obtained in a similar procedure as described for [**2a**]Cl with 4,-4'-diphenyl-2,2'-bipyridine instead of 4,-4'-di-*tert*-butyl-2,2'-bipyridine. Racemic Ir complex salt [**2c**]Cl was purified by recrystallization from

CH_2Cl_2-hexane solution (94% isolated yield).

Synthesis of non-racemic Ir complex salt [2]Cl. The preparation of the chiral auxiliary ligand and the optical resolution of the chiral Ir complexes were performed according to the literature (Supplementary Fig. 1).[3] The dinuclear Ir precursor complex $[Ir_2(ppy)_4(\mu\text{-}Cl)_2]$ (107 mg, 0.10 mmol), a chiral auxiliary ligand, (*S*)-2-(2'-hydroxyphenyl)-4-isopropyl-2-thiazoline (49.7 mg, 0.22 mmol) and Et_3N (278 μL, 2.0 mmol) were dissolved in CH_2Cl_2 (10 mL) and the mixture was heated at 40 °C for 6 h under N_2 atmosphere. After cooling to room temperature, the mixture was dried in vacuo to provide a yellow solid containing a mixture of Λ-(*S*)- and Δ-(*S*)-Ir complexes. The diastereomers were separated by standard silica-gel chromatography. The isolated diastereomer, Λ-(*S*)- and Δ-(*S*)-Ir complexes, were reacted with corresponding bpy ligands in MeOH in the presence of 5 equiv. of HCl to give enantiomerically pure Λ-[**2**]Cl and Δ-[**2**]Cl, respectively. The chiral Ir complex salts, Λ/Δ-[**2a**]Cl and Λ/Δ-[**2b**]Cl, were purified by recrystallization from $CHCl_3$-hexane and CH_2Cl_2-hexane solutions, respectively.

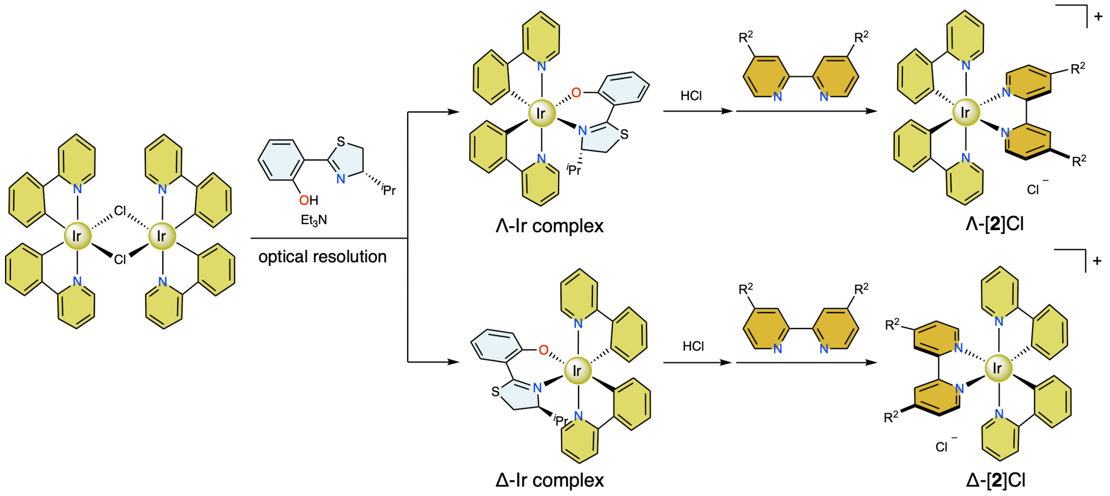

Supplementary Fig. 1 Schematic representation of auxiliary-mediated synthesis of enantiomerically pure Λ- and Δ- Ir complex salts.

Synthesis of racemic Ru complex salt [3a]Cl₂. Racemic Ru complex salt [**3a**]Cl_2 was synthesized according to an established method of similar Ru complexes.[4] $[Ru(bpy)_2Cl_2]$ (96.8 mg, 0.20 mmol) and 4,-4'-di-*tert*-butyl-2,2'-bipyridine (59.1 mg, 0.22 mmol) were dissolved in a mixture of ethanol (5 mL) and water (5 mL), and the solution was refluxed under N_2 atmosphere for 12 h. The reaction mixture was cooled to room temperature and the solvents were removed by evaporation. The solid was collected and purified by recrystallization from MeOH/Et_2O solution (62% isolated yield).

Synthesis of racemic Ru complex salt [3b]Cl₂. Racemic Ru complex salt [**3b**]Cl_2 was synthesized in a similar procedure of [**3a**]Cl_2 with 4,-4'-dimethyl-2,2'-bipyridine instead of 4,-4'-di-*tert*-butyl-2,2'-bipyridine. Racemic Ru complex salt [**3b**]Cl_2 was purified by recrystallization from MeOH/Et_2O solution (56% isolated yield).

General procedure for encapsulation experiments

To a wet chloroform solution (0.5 mL) of resorcin[4]arene **1** (6.6 µmol) was added Ir complex salt [**2**]X or Ru complex salt [**3**]X_2 (1.0 µmol). After the mixture was heated at 50 ºC for 1 h in the dark, formation of inclusion complexes (**1**)$_6$⊃([**2**]X) and (**1**)$_6$⊃([**3**]X_2) were confirmed by NMR spectroscopy.

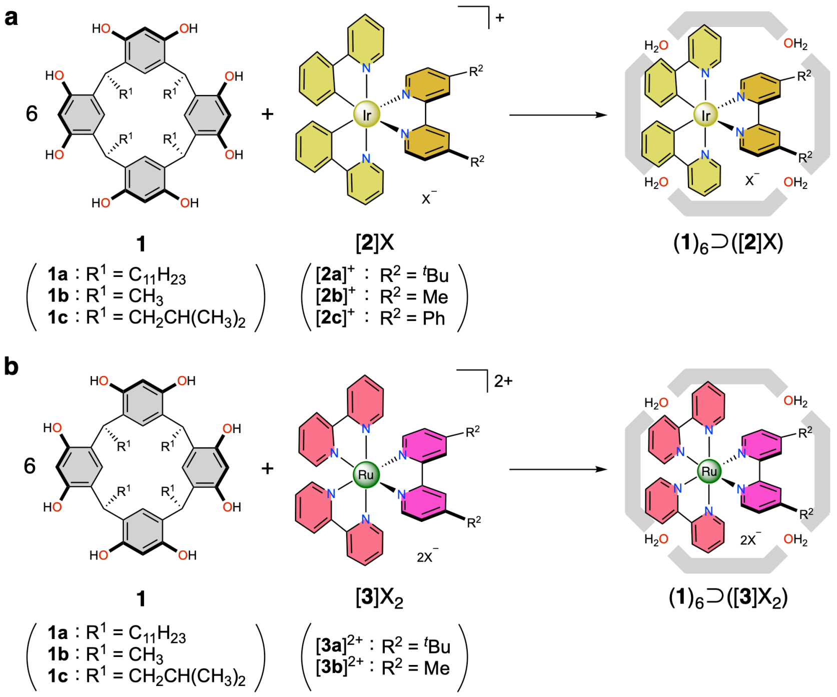

Supplementary Fig. 2 Schematic representation of encapsulation of **a** Ir complex [**2**]X (X = Cl, NO_3, PF_6), and **b** Ru complex [**3**]X_2 (X = Cl) within the resorcin[4]arene capsule.

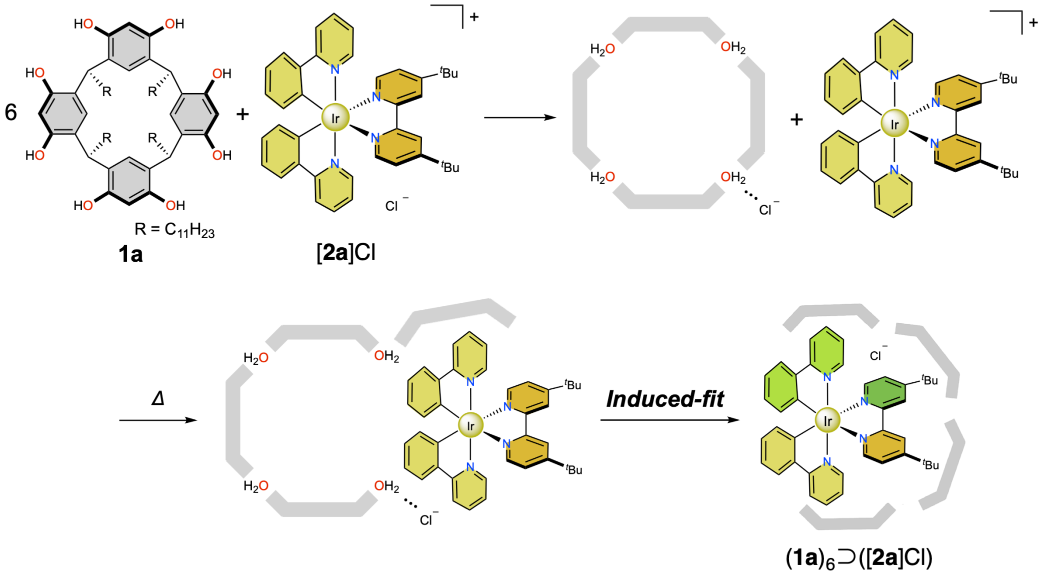

Supplementary Fig. 3 Plausible self-assembly and encapsulation mechanism of (**1a**)$_6$⊃([**2a**]Cl). After mixing the solutions of **1a** and [**2a**]Cl, the hexameric capsule initially captures a Cl anion via hydrogen bonding to a water molecule in the capsule framework to afford a mixture of anionic capsule and free Ir complex cation. Heating accelerates partial dissociation of a resorcin[4]arene monomer from the capsule.[5] The Ir complex cation is accommodated through the window of the capsule. As the size and shape of the guest is not adapted within the cavity of the symmetric hexameric capsule, a large structural change of the host framework occurs to give the distorted hexameric capsule around the Ir complex cation.

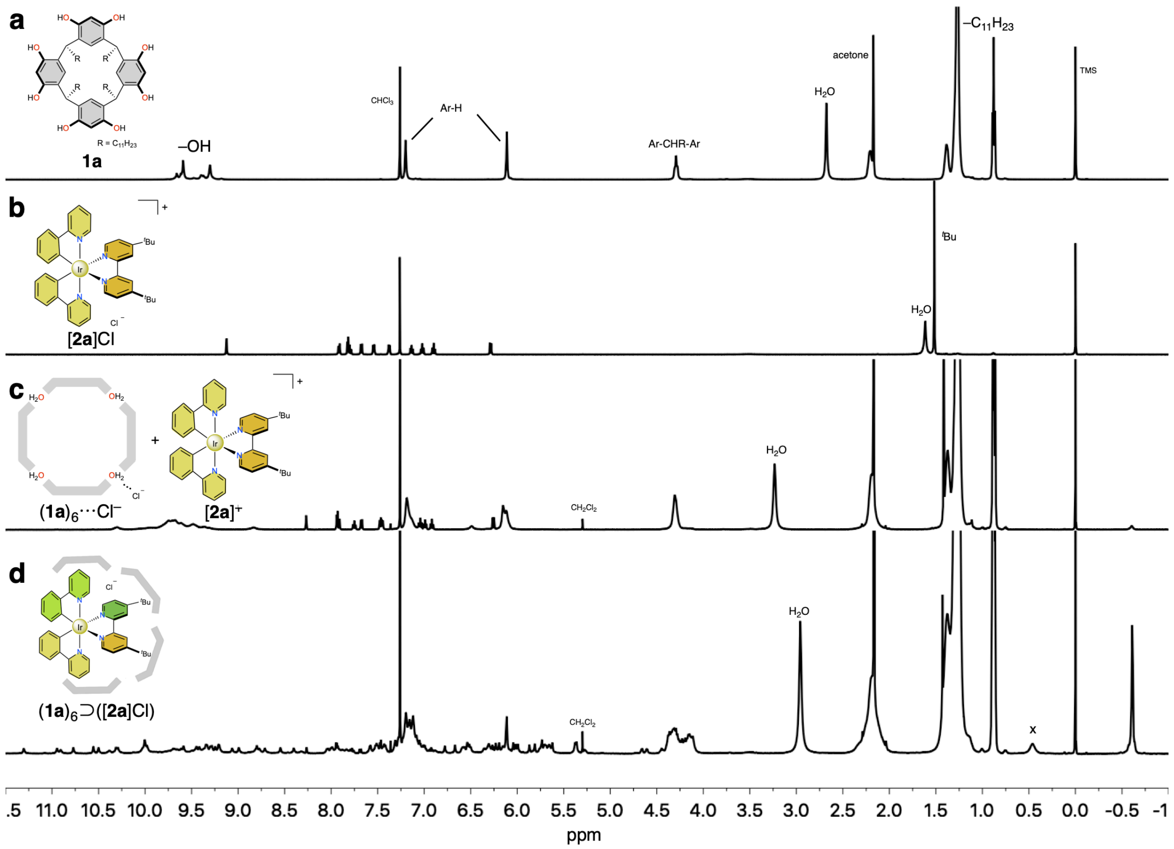

Supplementary Fig. 4 ^{1}H NMR spectra (500 MHz, $CDCl_3$, r.t.) of **a** resorcin[4]arene **1a**, **b** Ir complex [**2a**]Cl, **c** a mixture of (**1a**)$_6$ and [**2a**]Cl, and **d** the mixture after heating at 50 °C for 1 h.

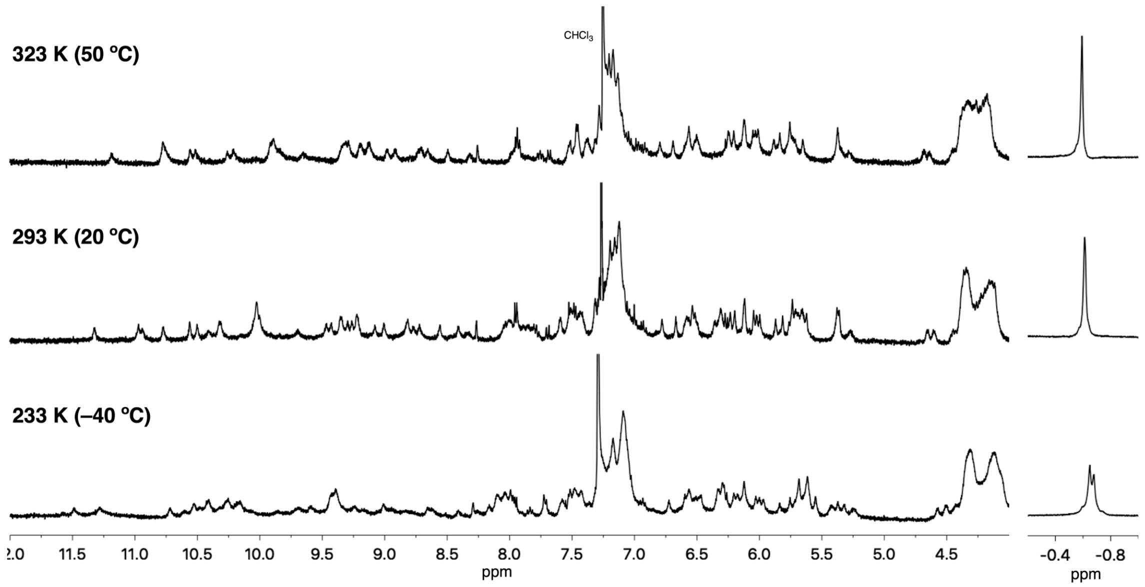

Supplementary Fig. 5 Variable temperature (VT) ^{1}H NMR spectra (400 MHz, $CDCl_3$) of (**1a**)$_6$⊃([**2a**]Cl). The NMR signals were still splitting even at 50 °C, suggesting that the symmetry-breaking structure is maintained in these conditions.

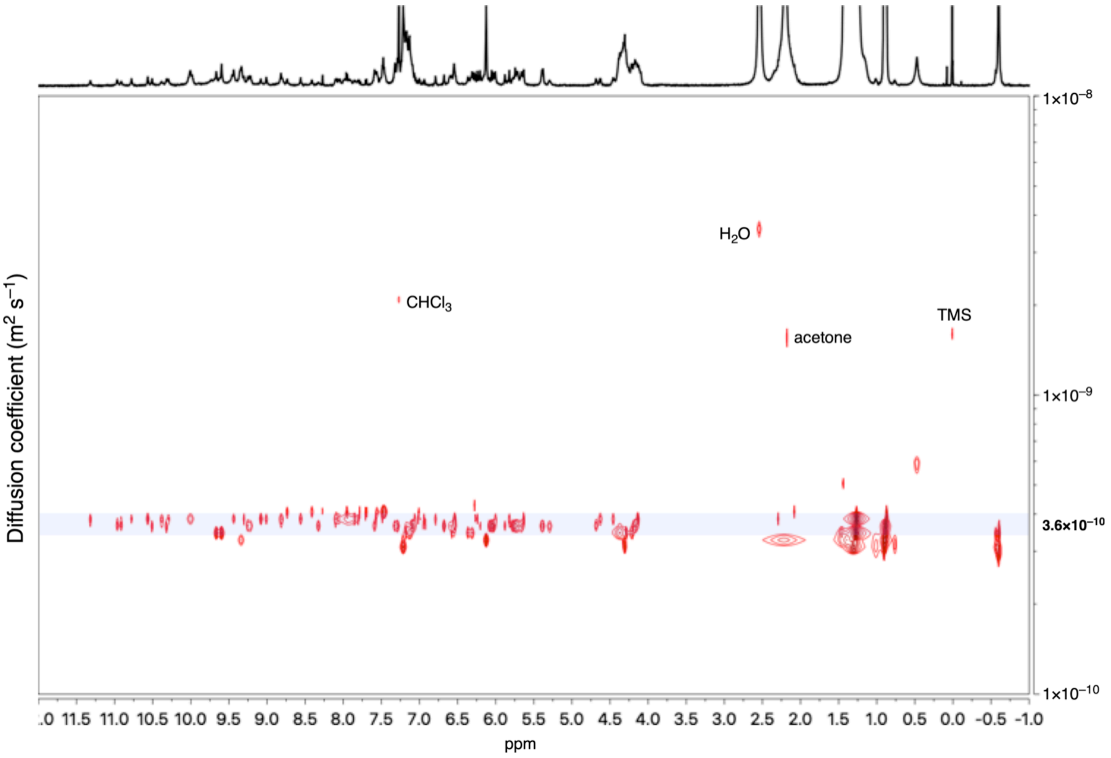

Supplementary Fig. 6 DOSY NMR spectrum (500 MHz, $CDCl_3$, r.t.) of $(\mathbf{1a})_6\supset([\mathbf{2a}]Cl)$.

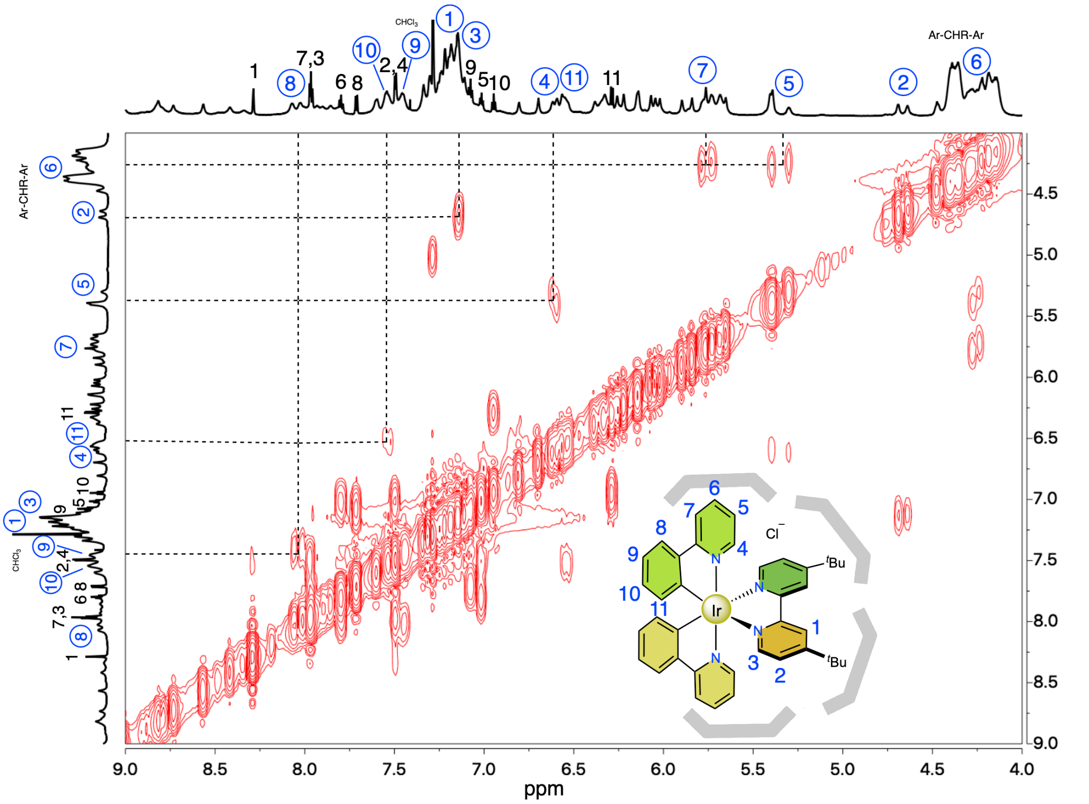

Supplementary Fig. 7 ^1H–^1H COSY spectrum ($CDCl_3$, r.t.) of $(\mathbf{1a})_6\supset([\mathbf{2a}]Cl)$. The signals marked with black and blue encircled numbers were assigned to the protons of free $[\mathbf{2a}]^+$ and encapsulated $[\mathbf{2a}]^+$, respectively.

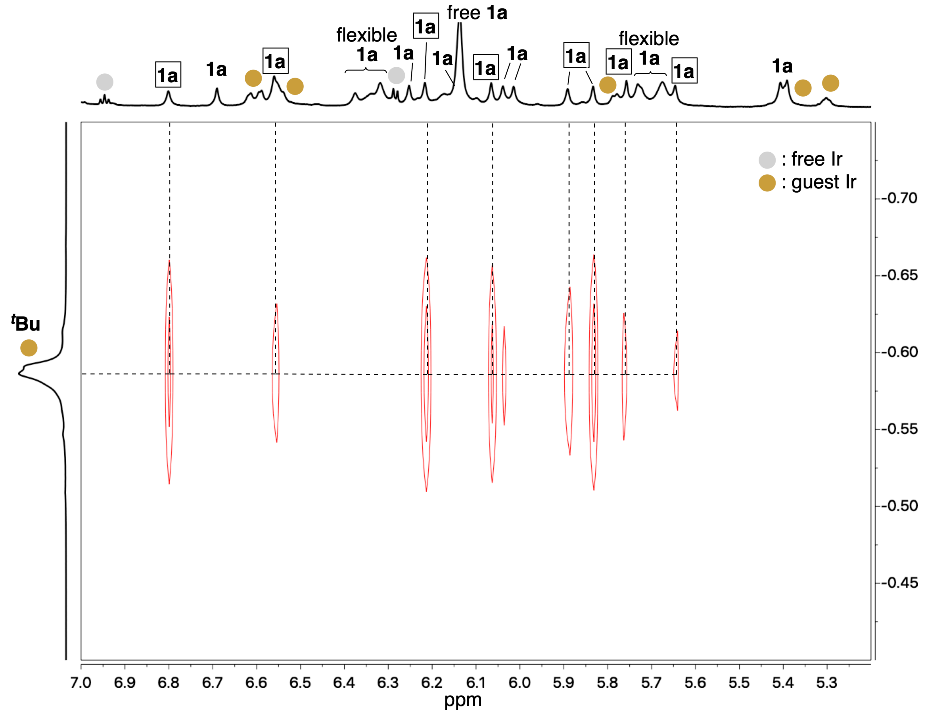

Supplementary Fig. 8 ^{1}H–^{1}H NOESY spectrum ($CDCl_3$, r.t.) of $(\mathbf{1a})_6\supset([\mathbf{2a}]Cl)$. Strong 8 cross peaks were observed between the ^{t}Bu protons of encapsulated $[\mathbf{2a}]^{+}$ and aromatic protons of the distorted hexameric capsule, indicating that two resorcin[4]arene units were located around the ^{t}Bu groups.

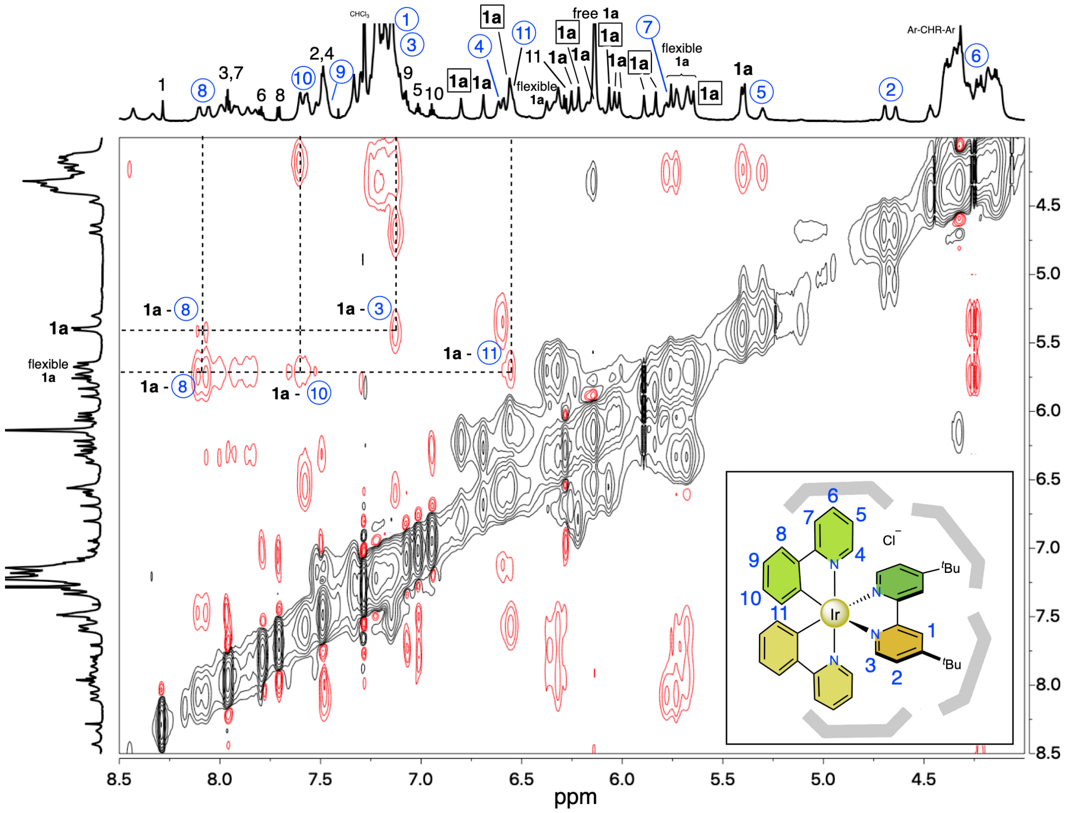

Supplementary Fig. 9 ROESY spectrum (800 MHz, $CDCl_3$, 25 ºC) of $(\mathbf{1a})_6\supset([\mathbf{2a}]Cl)$. The black and blue encircled numbers were assigned to the protons of free and encapsulated $[\mathbf{2a}]^{+}$, respectively. The signals of resorcin[4]arenes showing strong NOE with the ^{t}Bu proton of $[\mathbf{2a}]^{+}$ were marked in a square.

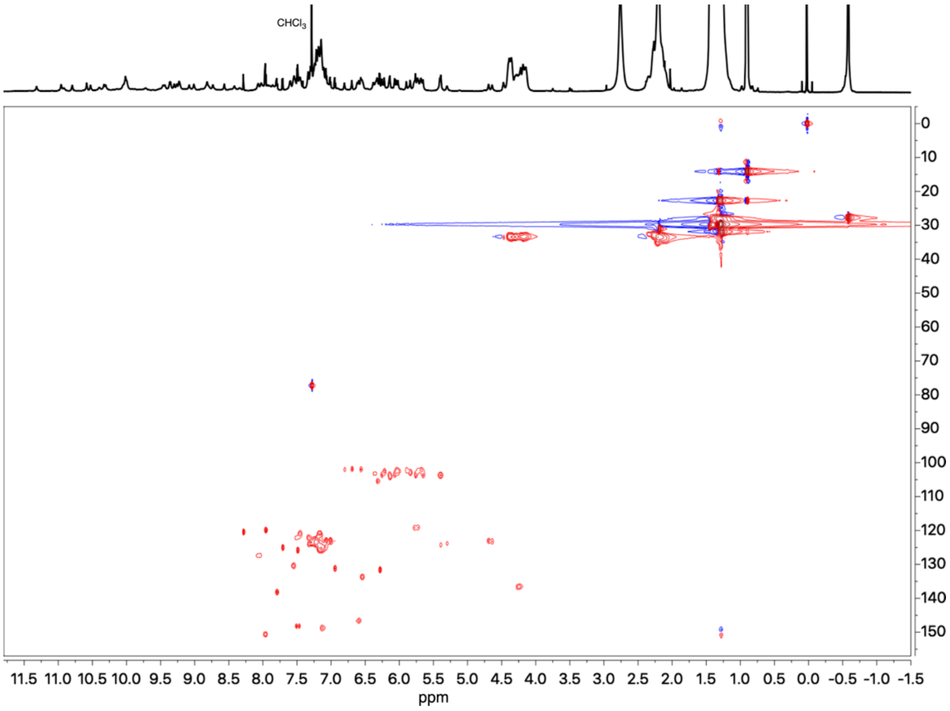

Supplementary Fig. 10 ^1H–^{13}C HSQC spectrum ($CDCl_3$, r.t.) of (**1a**)$_6$⊃([**2a**]Cl).

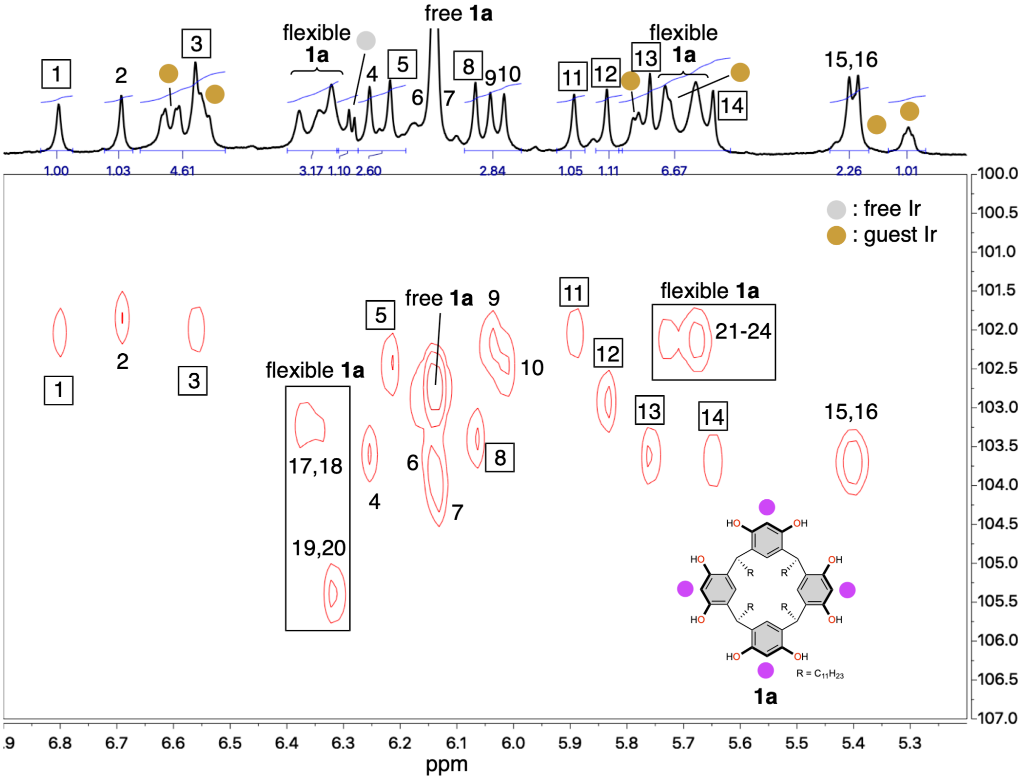

Supplementary Fig. 11 Enlarged ^1H–^{13}C NMR spectrum ($CDCl_3$, 25 ºC) around aromatic protons **1a** in host–guest complex [(**1a**)$_6$⊃[**2a**]Cl]. The 24 cross peaks derived from the resorcin[4]arene marked with the magenta filled circle (●) were observed. The signals of resorcin[4]arenes showing strong NOE with the ^{t}Bu proton of encapsulated [**2a**]$^+$ were marked in a square. This result suggests that six resorcin[4]arene units formed a defined C_1 symmetric hexameric capsule.

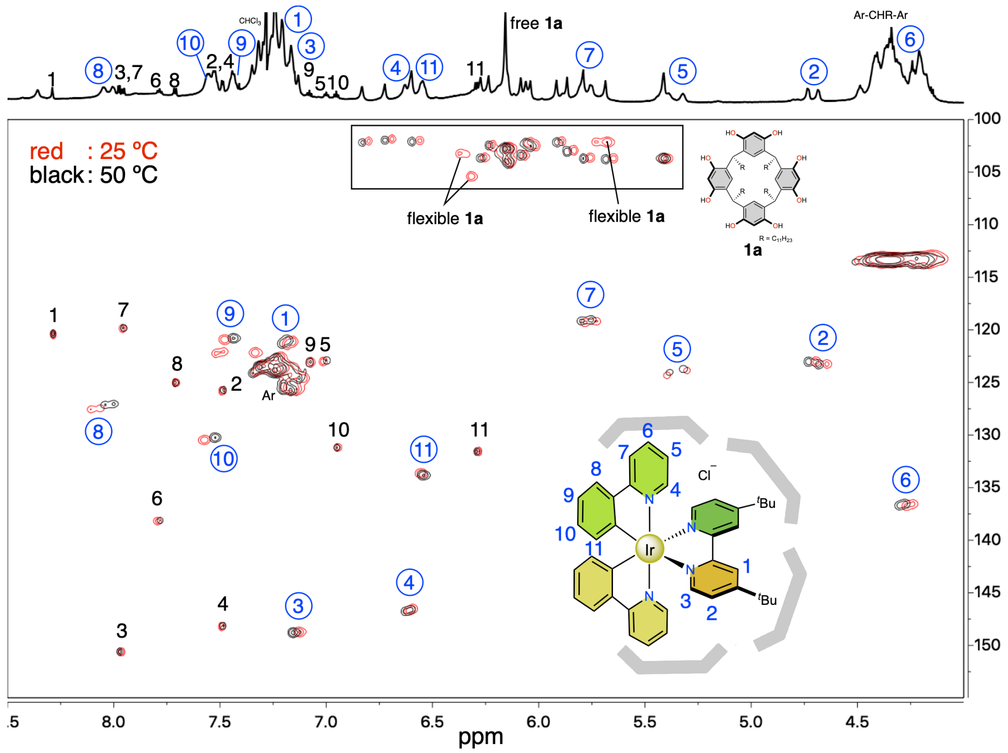

Supplementary Fig. 12 ^{1}H–^{13}C HSQC spectrum of $(\mathbf{1a})_6\supset([\mathbf{2a}]Cl)$ recorded at 50 ºC (black), superimposed with the spectrum at 25 ºC (red). The cross peaks marked with black and blue encircled numbers were assigned to the protons of free $[\mathbf{2a}]^+$ and encapsulated $[\mathbf{2a}]^+$, respectively. The broad peaks assigned to the flexible **1a** disappeared at 50 ºC, while other peaks were still sharp and slightly shifted. This result suggests that two in the distorted hexameric resorcin[4]arene capsule are relatively flexible around the Ir complex guest, compared with other four units.

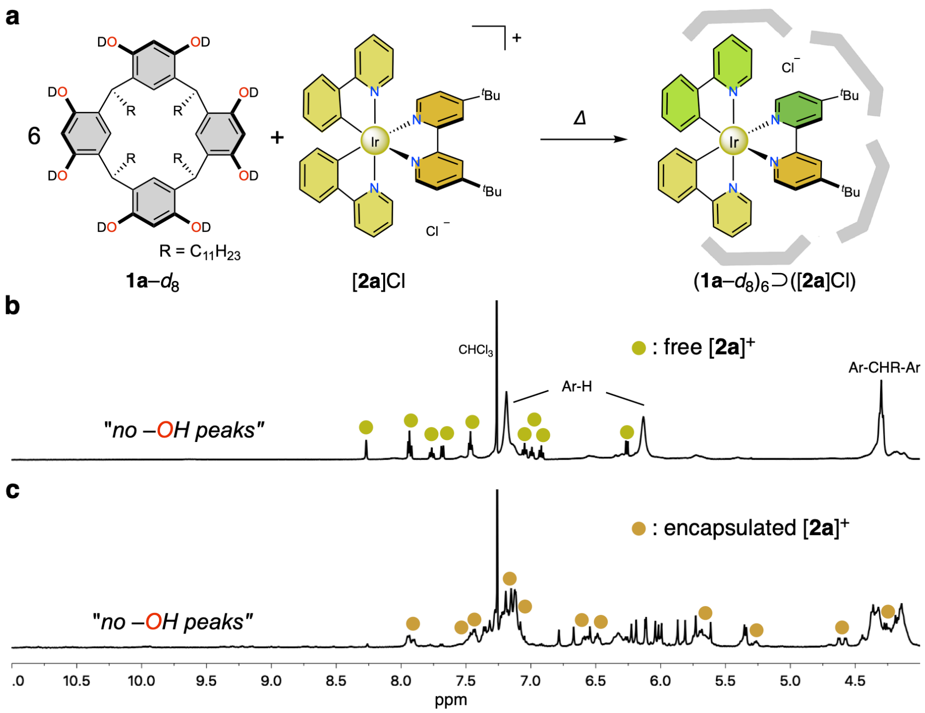

Supplementary Fig. 13 **a** Schematic representation of the formation of $(\mathbf{1a}\text{–}d_8)_6\supset([\mathbf{2a}]Cl)$. **b,c** ^{1}H NMR spectra (500 MHz, $CDCl_3$, r.t.) of **b** a mixture of $(\mathbf{1a}\text{–}d_8)_6$ and $[\mathbf{2a}]Cl$, and **c** the mixture after heating at 50 ºC for 1 h.

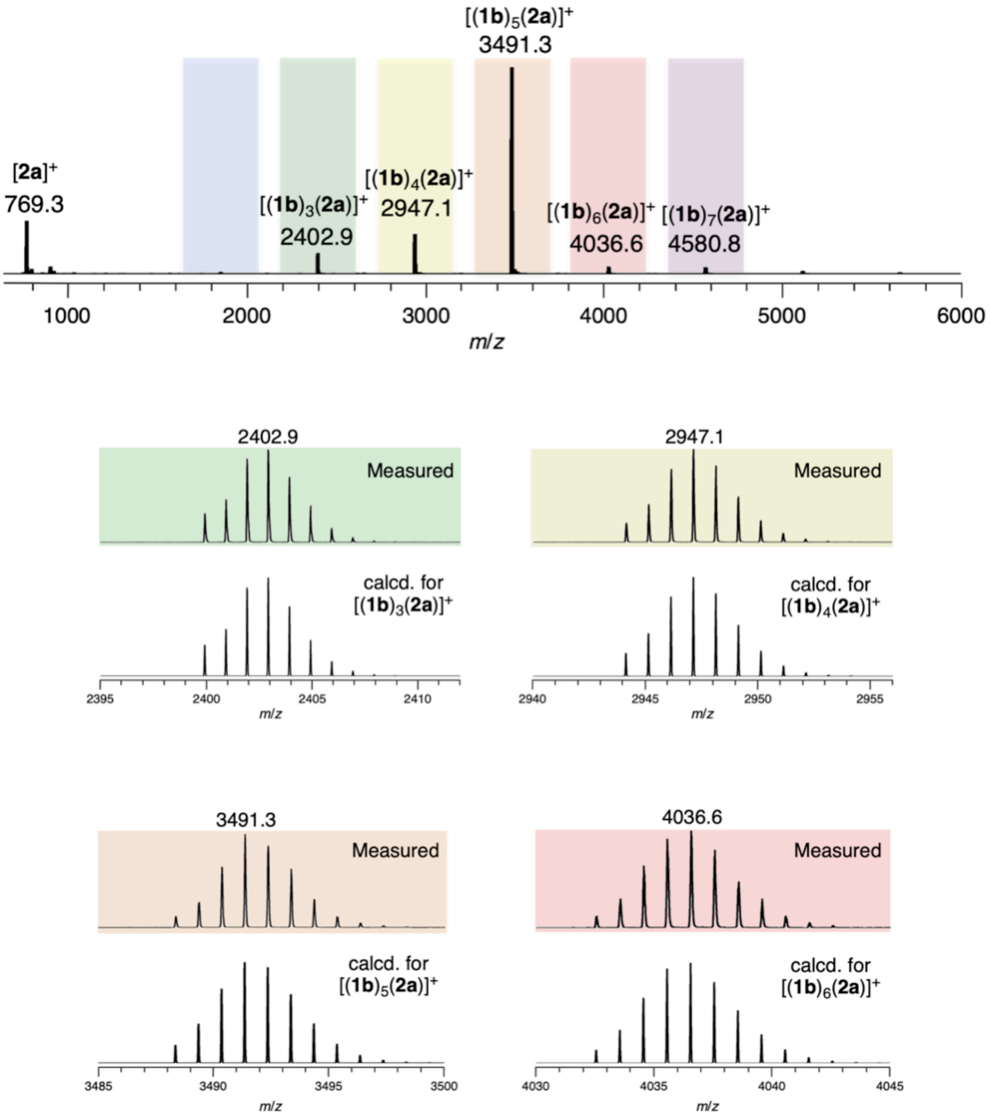

Supplementary Fig. 14 ESI-MS spectra of a mixture of resorcin[4]arene **1b** (R^1 = Me) and Ir complex salt [**2a**]Cl in $CHCl_3$/acetone (2:1, v/v). The most intense signal was observed at *m*/*z* 3491.3, assigned to the host–guest complex in 5:1 stoichiometry. While this result seems inconsistent with the solution structure of stoichiometry (**1a**)$_6$⊃([**2a**]Cl), the different environments the fragile assembly is exposed to can serve as explanation. In the mass spectrometry analysis, the water molecules joining the six resorcin[4]arene units via multiple hydrogen-bonds in solution are expelled during the gas-phase transfer into the vacuum system, as evident from the lack of water adducts. Therefore, the distorted hydrogen-bonded hexameric capsule can not be maintained and partial dissociation is promoted to a more compact complex in the ESI-MS study.

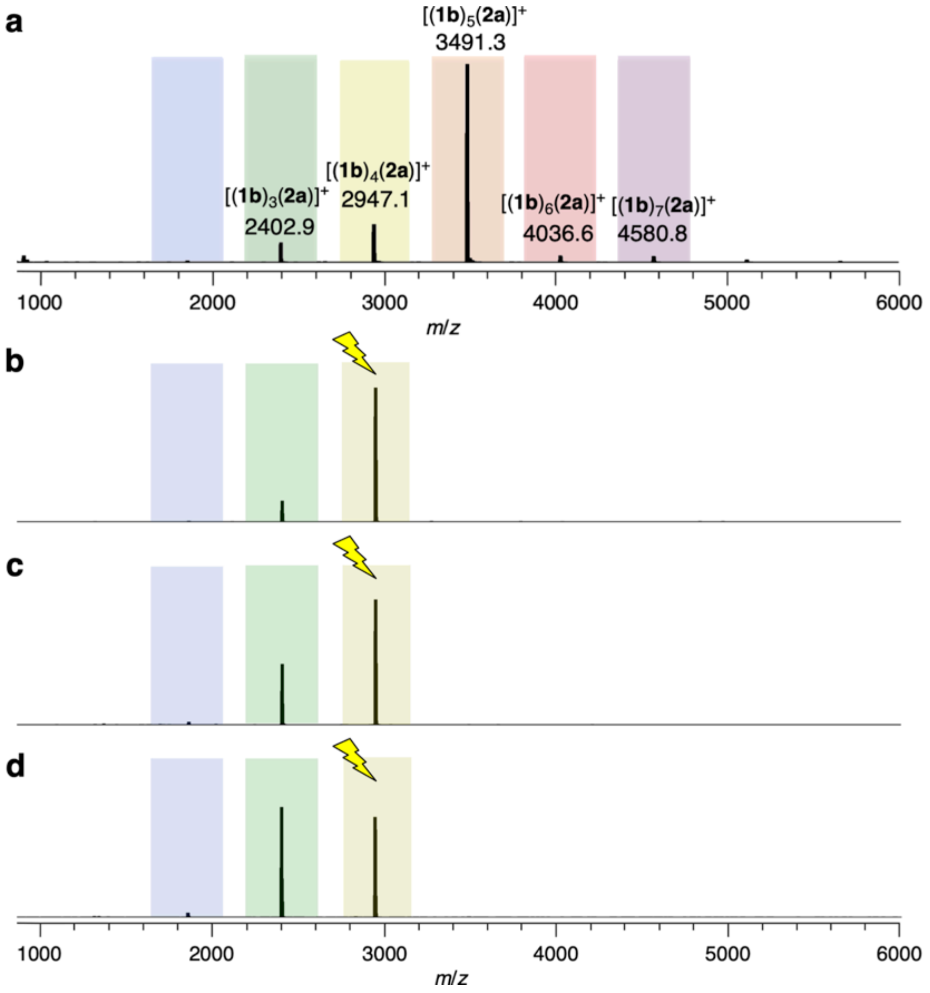

Supplementary Fig. 15 a ESI-MS spectra of a mixture of resorcin[4]arene **1b** (R^1 = Me) and Ir complex salt [**2a**]Cl in $CHCl_3$/acetone (2:1, v/v). **b–d** ESI tandem mass spectra of $[(\mathbf{1b})_4(\mathbf{2a})]^+$ (m/z : 2947.1) with the collision energy at **b** 10 eV, **c** 15 eV, and **d** 20 eV. Due to detection limit of the ESI-timsTOF mass spectrometer, the ESI tandem mass spectra were measured for $[(\mathbf{1b})_4(\mathbf{2a})]^+$. Higher collision energy promotes consecutive losses of a resorcin[4]arene unit from the parent ion.

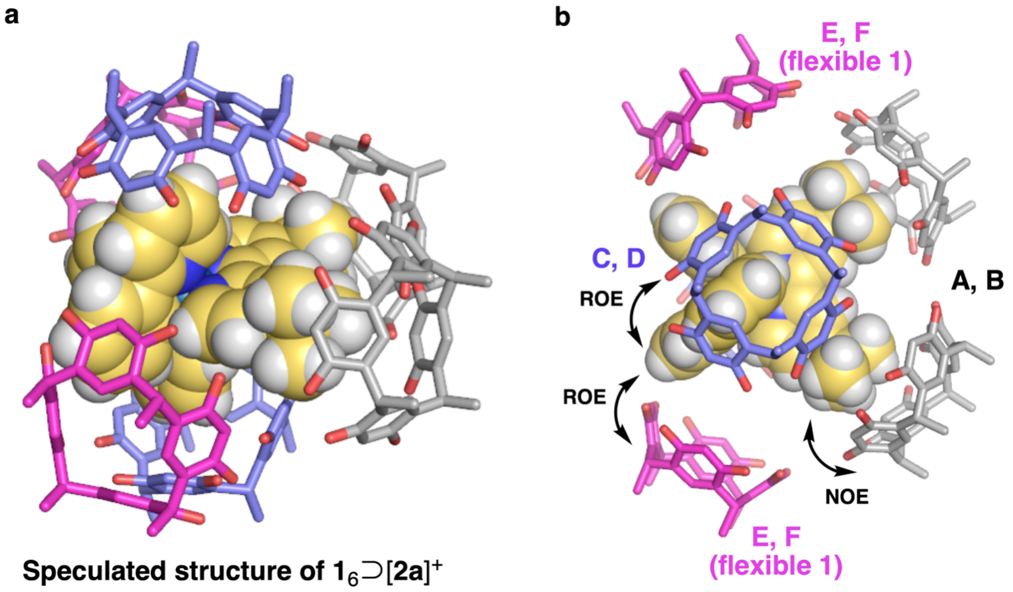

Supplementary Fig. 16 A modeling structure of $\mathbf{1}_6 \supset [\mathbf{2a}]^+$: **a** Front view, and **b** top view of the assembly, based on the 2D NMR results and X-ray structure of $\mathbf{1c}_3 \supset [\mathbf{3a}]^{2+}$. As the solution structure of assembly still involves uncertainty, water molecules and counter anion in the assembly cannot be included in this model. Nevertheless, this model structure roughly shows a relative orientation of the guest and hosts in solution.

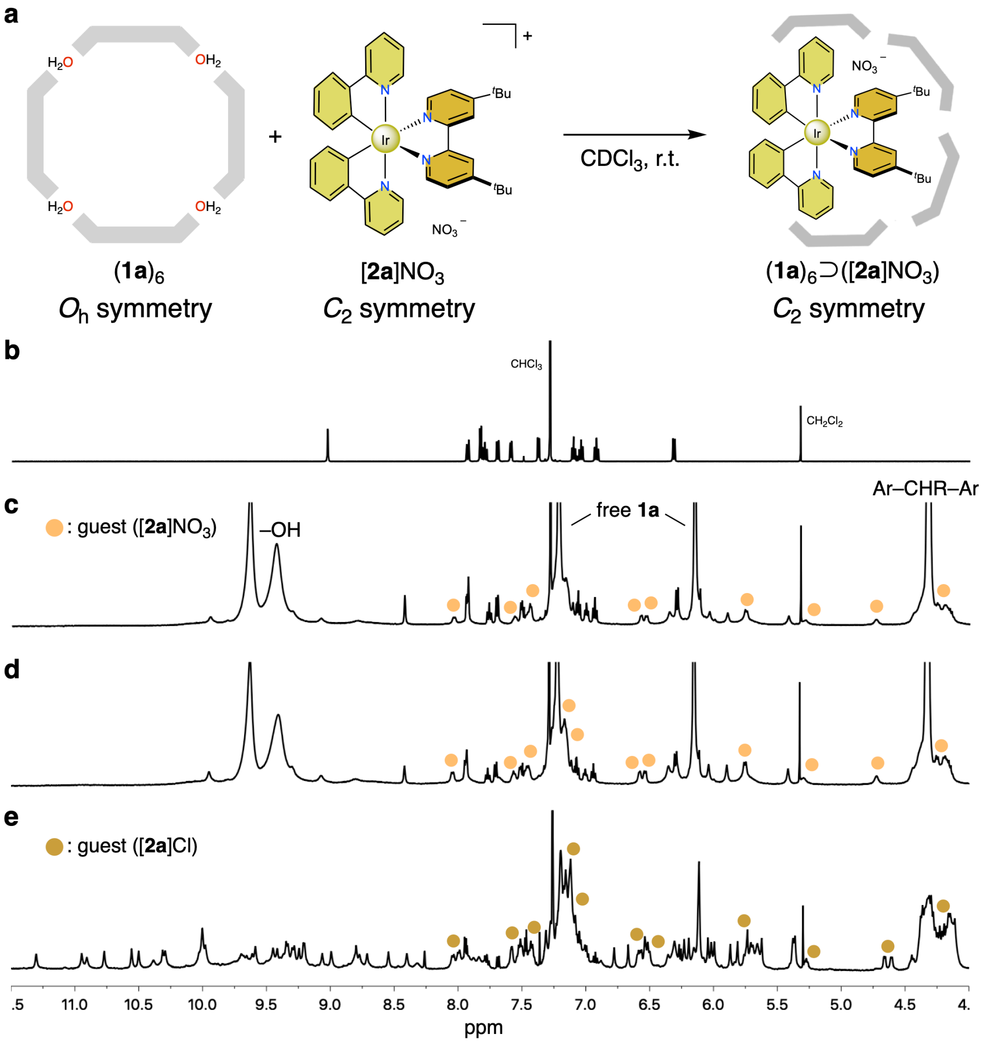

Supplementary Fig. 17 **a** Schematic representation of the assembly of [**2a**]NO_3. **b–e** ^{1}H NMR spectra (500 MHz, $CDCl_3$, r.t.) of **b** Ir complex [**2a**]NO_3, **c** a mixture of (**1a**)$_6$ and [**2a**]NO_3, **d** a mixture after 1 h at 50 ºC, and **e** the symmetry-breaking assembly (**1a**)$_6$⊃([**2a**]Cl). The NO_3 salt was encapsulated without heating the mixture to give symmetric host–guest complex (**1a**)$_6$⊃([**2a**]NO_3) in NMR time-scale. These results indicate that NO_3 anion weaken the hydrogen-bond network of the hexameric capsule to increase flexibility of **1a**.

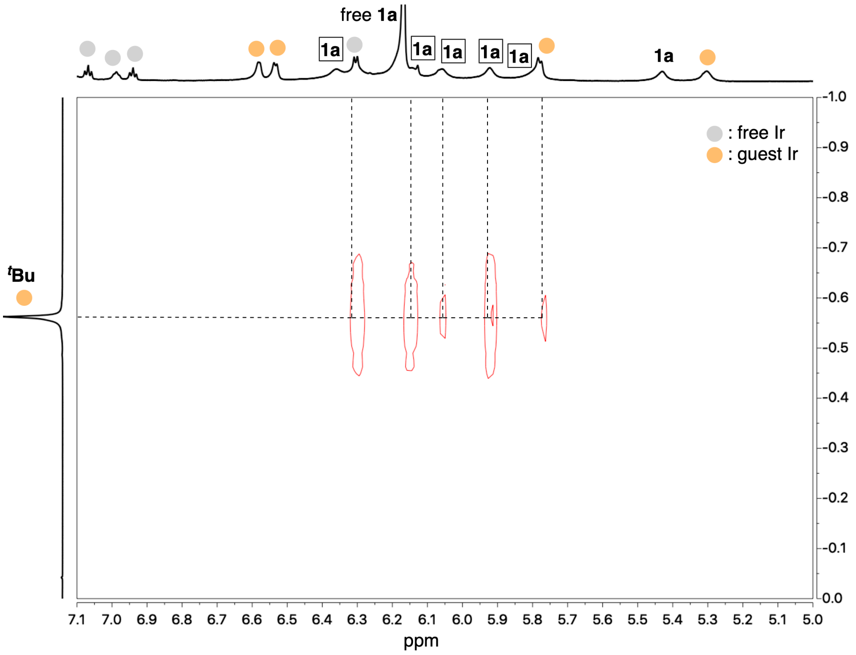

Supplementary Fig. 18 ^1H–^1H NOESY spectrum ($CDCl_3$, r.t.) of (**1a**)$_6$⊃([**2a**]NO_3). Cross peaks were observed between the ^{t}Bu protons of encapsulated [**2a**]$^+$ and aromatic protons of the distorted hexameric capsule.

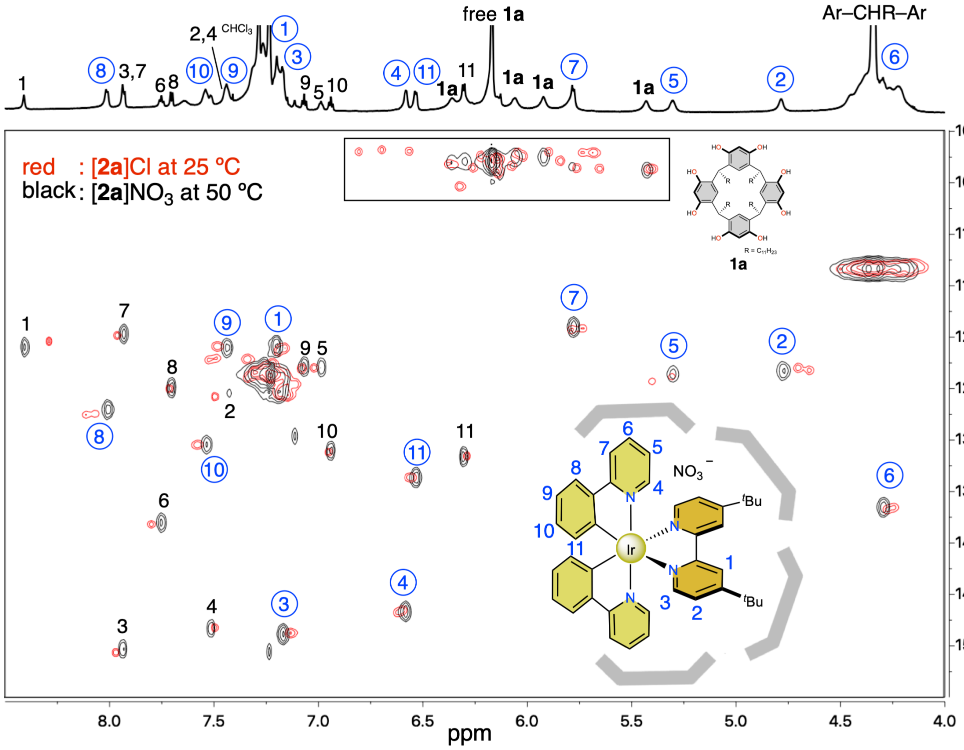

Supplementary Fig. 19 ^1H–^{13}C HSQC spectrum of (**1a**)$_6$⊃([**2a**]NO_3) recorded at 50 ºC (black), superimposed with the spectrum of (**1a**)$_6$⊃([**2a**]Cl) at 25 ºC (red). The cross peaks marked with black and blue encircled numbers were assigned to the protons of free [**2a**]$^+$ and encapsulated [**2a**]$^+$, respectively. Similar chemical shifts of encapsulated [**2a**]NO_3 were observed to those of [**2a**]Cl, indicating that both guest salts have similar configurations within the distorted hexameric capsule. In particular, the peak splitting pattern of the guests suggests that the C_2 molecular symmetry of the guest was kept even after encapsulation on the NMR time-scale.

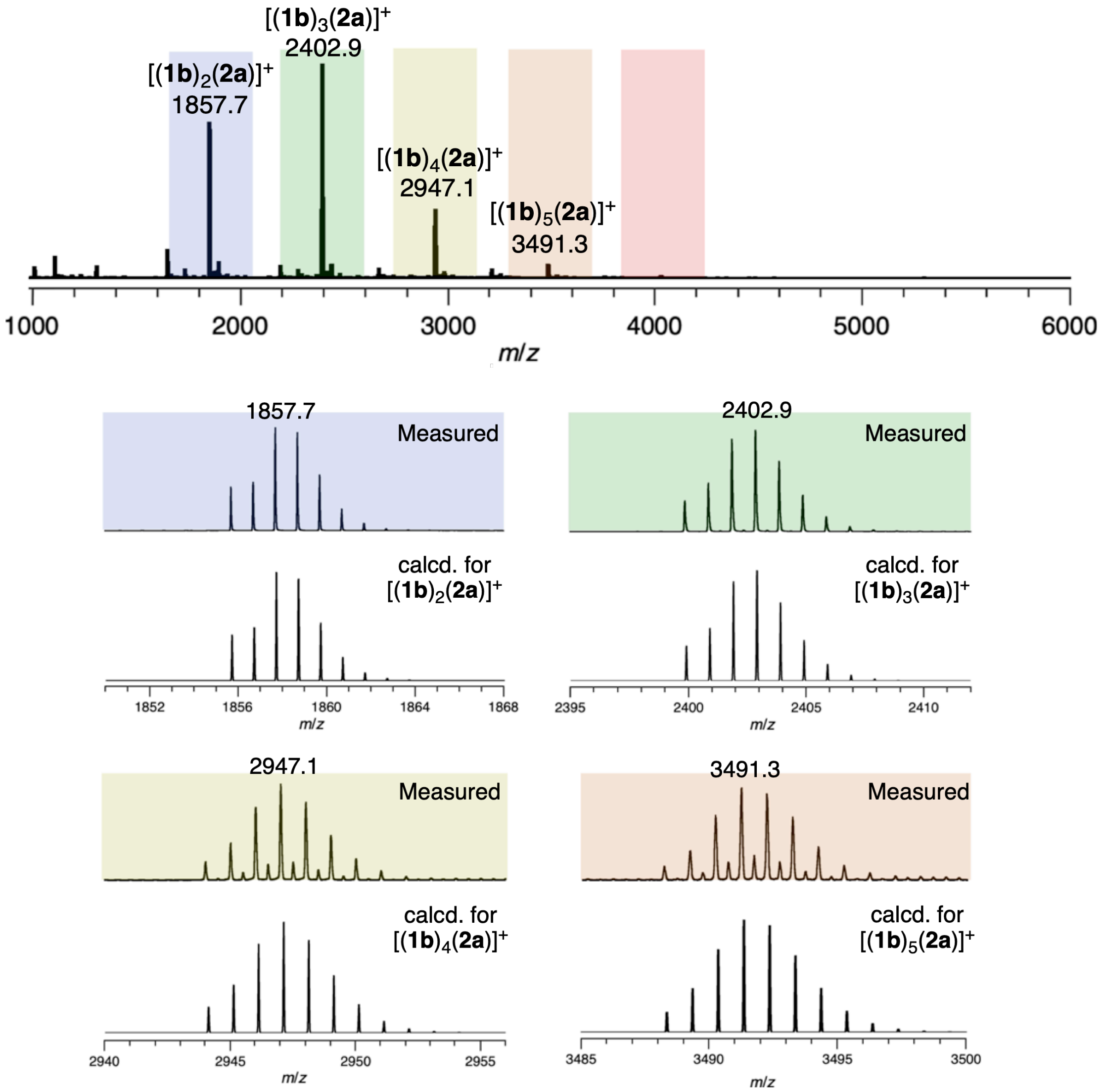

Supplementary Fig. 20 ESI-MS spectra of a mixture of resorcin[4]arene **1b** (R^1 = Me) and Ir complex salt [**2b**]NO_3 in $CHCl_3$/acetone (2:1, v/v). The effective template effect from the complex cation afforded supramolecular Ir complexes encapsulated by resorcin[4]arene oligomers in the gas-phase. However, the template effect of the NO_3 salt is not strong enough to stabilize the larger assemblies in the gas-phase.

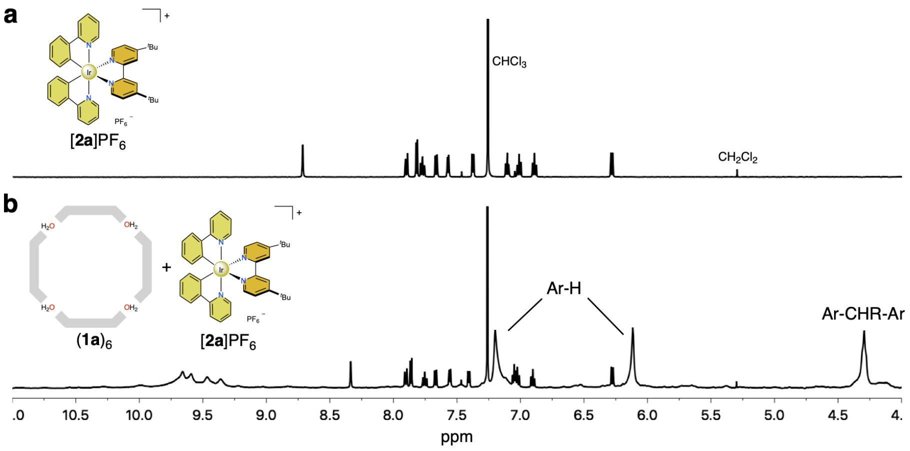

Supplementary Fig. 21 Enlarged ^{1}H NMR spectra (500 MHz, $CDCl_3$, r.t.) of **a** Ir complex [**2a**]PF_6, and **b** a mixture of (**1a**)$_6$ and [**2a**]PF_6 after heating at 50 °C for 1 h. The signals of the mixture did not change after heating, indicating that Ir complex salt [**2a**]PF_6 was not encapsulated and that the nature of the counter anion plays an important role in the encapsulation process.

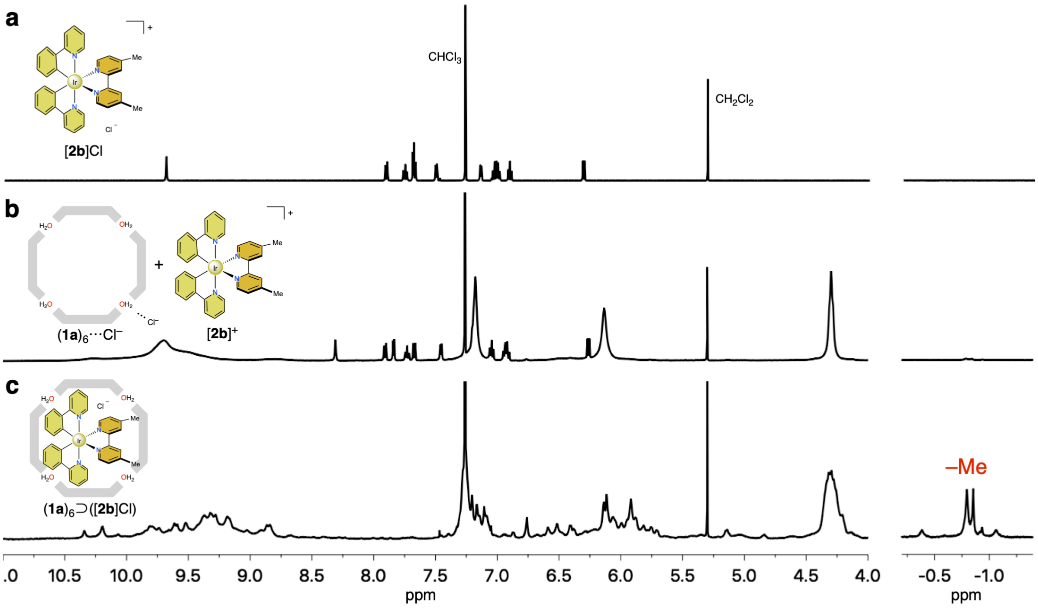

Supplementary Fig. 22 ^{1}H NMR spectra (500 MHz, $CDCl_3$, r.t.) of **a** Ir complex [**2b**]Cl, **b** a mixture of (**1a**)$_6$ and [**2b**]Cl, and **c** the mixture after heating at 50 °C for 1 h. The signals were broadened and shifted to up-field region after host–guest formation, indicating that the Ir complex salt was encapsulated within the resorcin[4]arene capsule.

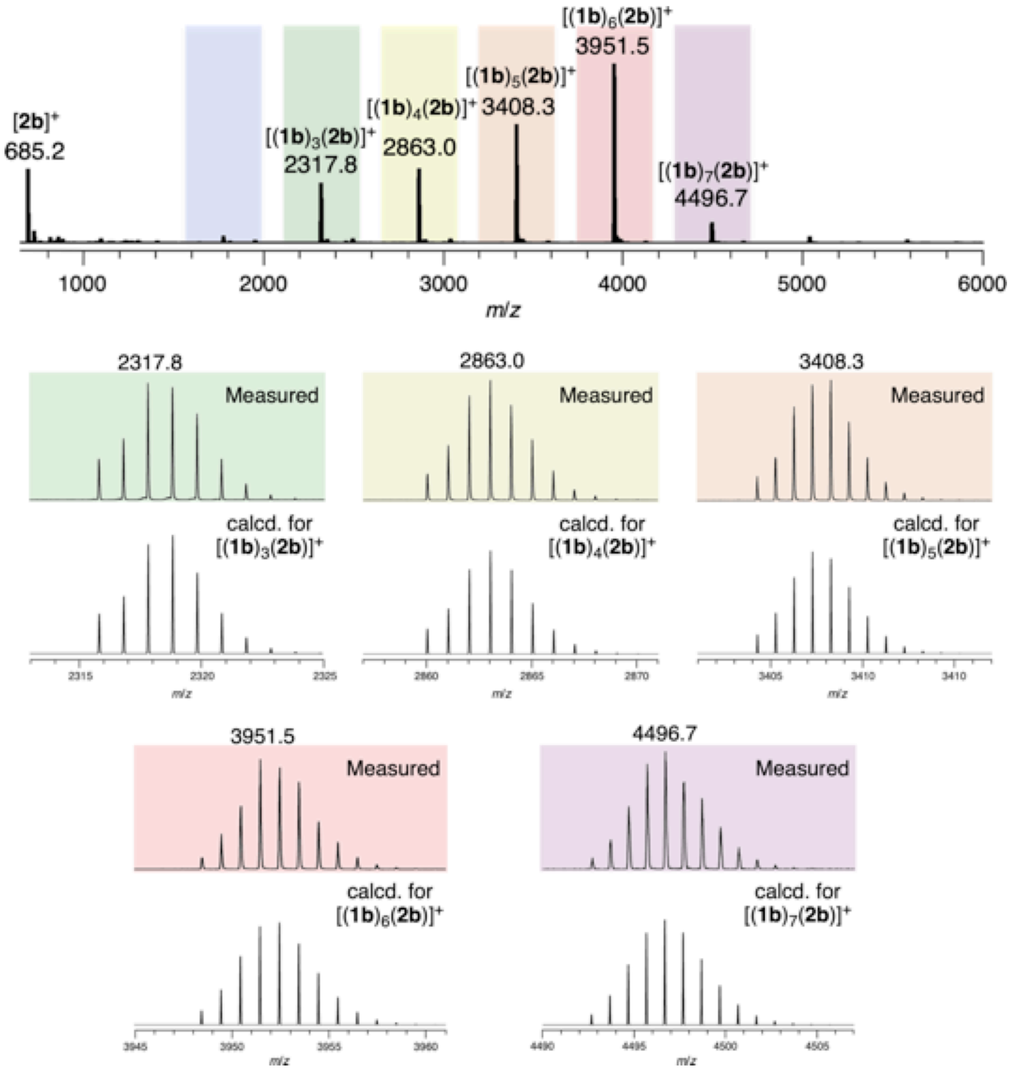

Supplementary Fig. 23 ESI-MS spectra of a mixture of resorcin[4]arene **1b** (R^1 = Me) and Ir complex salt [**2b**]Cl in $CHCl_3$/acetone (2:1, v/v). Effectively templated by the smaller complex cation, mainly large supramolecular assemblies of up to seven resorcin[4]arenes surrounding the Ir complex were observed in the gas-phase.

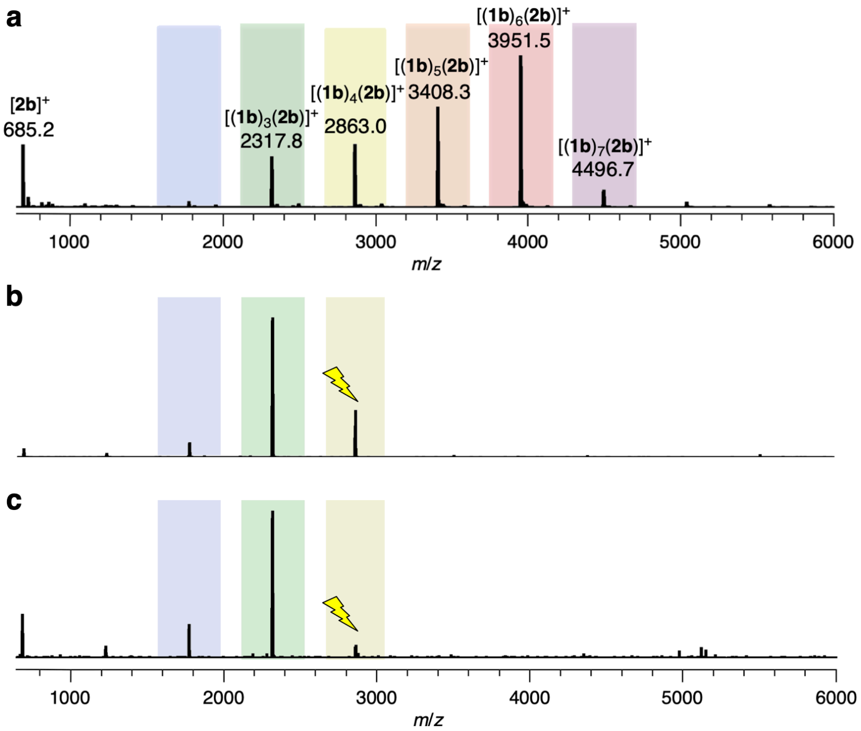

Supplementary Fig. 24 **a** ESI-MS spectra of a mixture of resorcin[4]arene **1b** (R^1 = Me) and Ir complex salt [**2b**]Cl in $CHCl_3$/acetone (2:1, v/v). **b–c** ESI tandem mass spectra of $[(\mathbf{1b})_4(\mathbf{2b})]^+$ (m/z : 2863.0) with the collision energy at **b** 1 eV, and **c** 10 eV. Due to detection limit of the ESI-timsTOF mass spectrometer, the ESI tandem mass spectra were measured for $[(\mathbf{1b})_4(\mathbf{2b})]^+$. Higher collision energy promotes consecutive monomer losses from the parent ion.

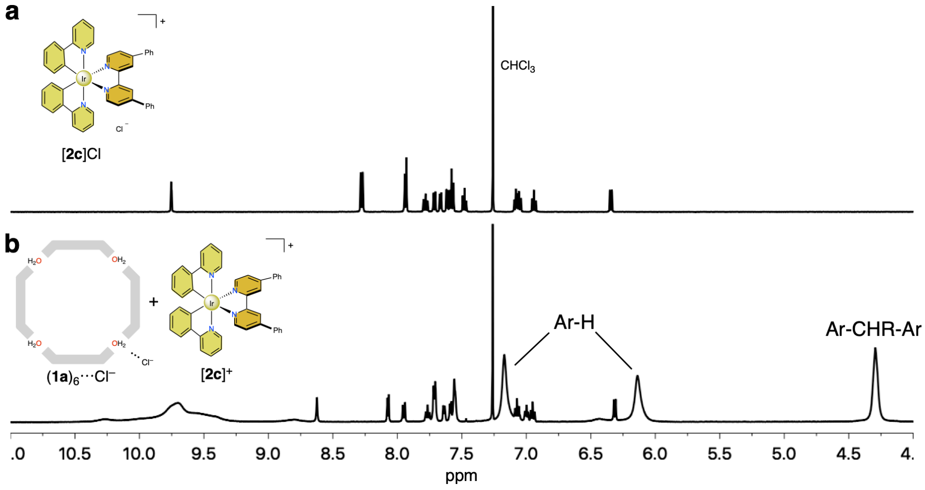

Supplementary Fig. 25 ^{1}H NMR spectra (500 MHz, $CDCl_3$, r.t.) of **a** Ir complex [**2c**]Cl, and **b** a mixture of $(\mathbf{1a})_6$ and [**2c**]Cl after heating at 50 ºC for 1 h. The signals of the mixture did not change after heating, indicating that the very spacious Ir complex salt [**2c**]Cl was not encapsulated.

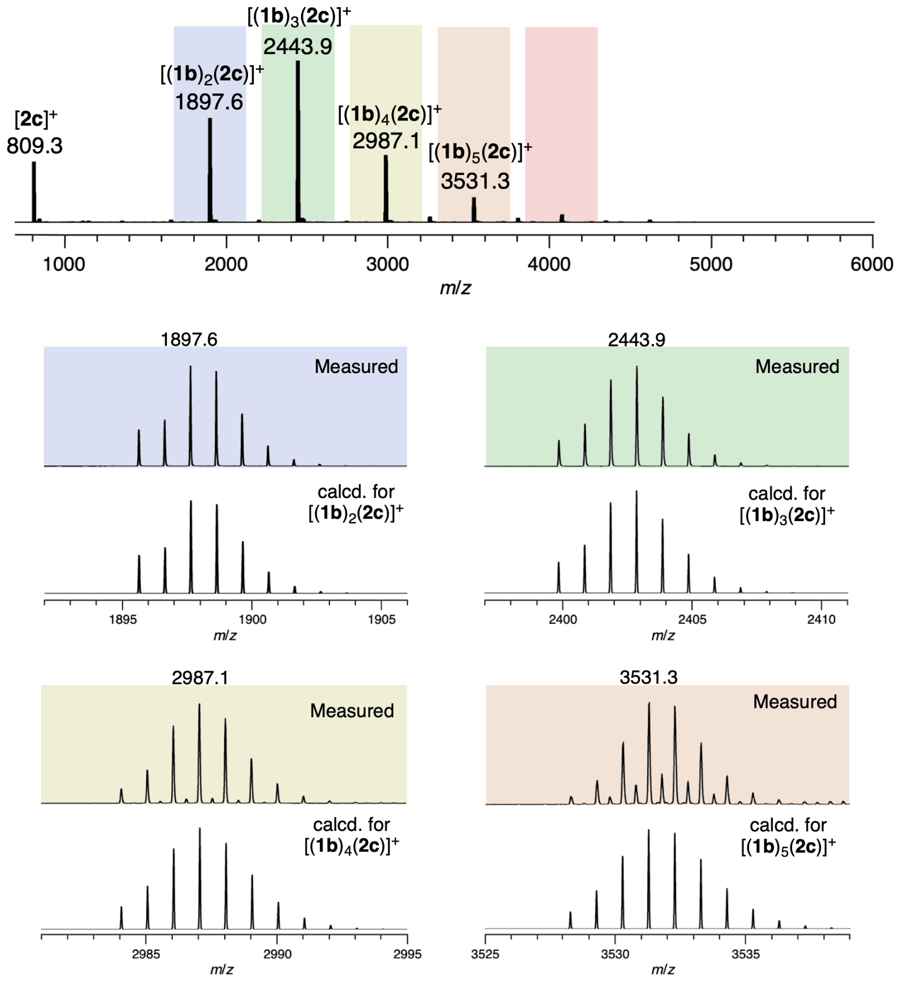

Supplementary Fig. 26 ESI-MS spectra of a mixture of resorcin[4]arene **1b** (R^1 = Me) and Ir complex salt [**2c**]Cl in $CHCl_3$/acetone (2:1, v/v). Although Ir complex salt [**2c**]Cl was not encapsulated within the resorcin[4]arene oligomer in solution, the effective template effect offered by the complex cation stabilizes a range of supramolecular assemblies between the Ir complex and resorcin[4]arene oligomers in the gas-phase. However, owing to the large size of the complex, exceeding the dimensions of the hexameric capsule, only smaller resorcin[4]arene oligomers were found to be stabilized.

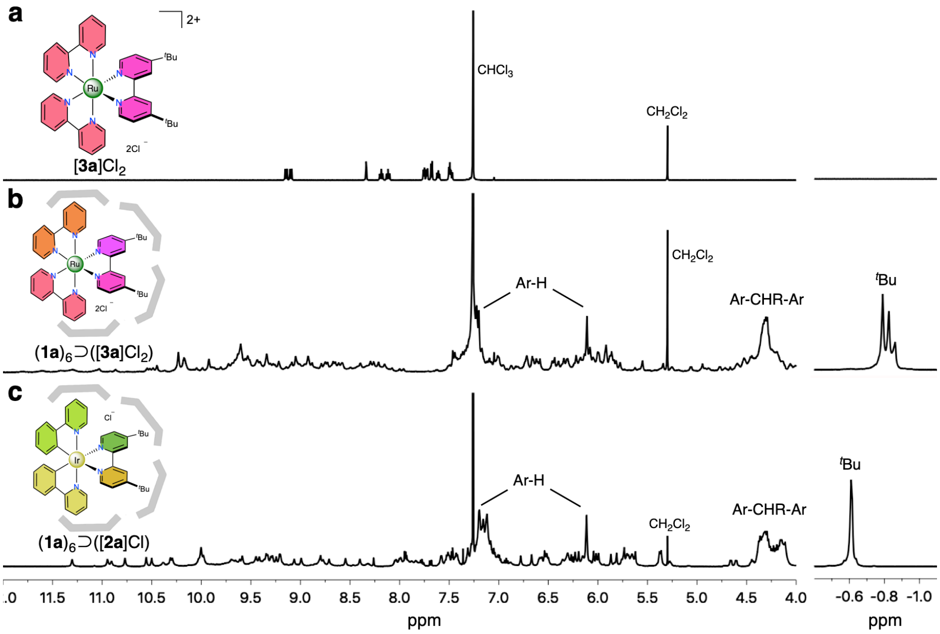

Supplementary Fig. 27 ^{1}H NMR spectra (500 MHz, $CDCl_3$, r.t.) of **a** [**3a**]Cl_2, **b** a mixture of (**1a**)$_6$ and [**3a**]Cl_2 after heating at 50 ºC for 1 h, and **c** symmetry-breaking assembly (**1a**)$_6$⊃([**2a**]Cl). The ^{1}H NMR spectrum of the mixture of (**1a**)$_6$ and [**3a**]Cl_2 also showed splitting signals of the assembly, suggesting a formation of symmetry-breaking host–guest complex (**1a**)$_6$⊃([**3a**]Cl_2). Notably, three ^{t}Bu signals derived from encapsulated [**3a**]$^{2+}$ were observed at –0.8 ppm, presumably owing to slow rotation of ^{t}Bu groups within the capsule on the NMR time-scale or existence of host–guest complexes with different stoichiometry (**1a**)$_n$⊃([**3a**]Cl_2) (n = 5–7) in solution.

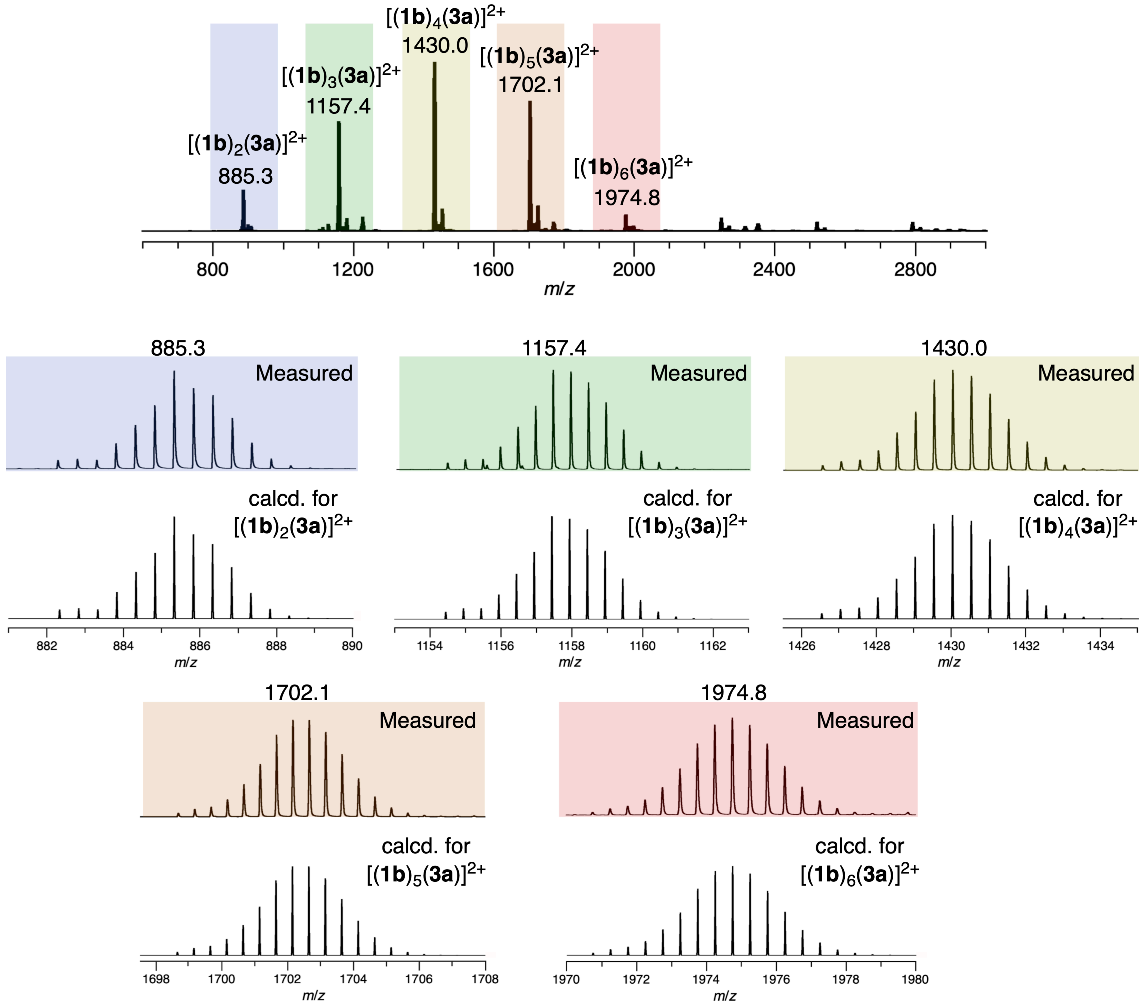

Supplementary Fig. 28 ESI-MS spectra of a mixture of resorcin[4]arene **1b** (R^1 = Me) and Ru complex salt [**3a**]Cl_2 in $CHCl_3$/acetone (2:1, v/v). The effective template effect from the complex cation affords supramolecular Ru complexes encapsulated by resorcin[4]arene oligomers in the gas-phase.

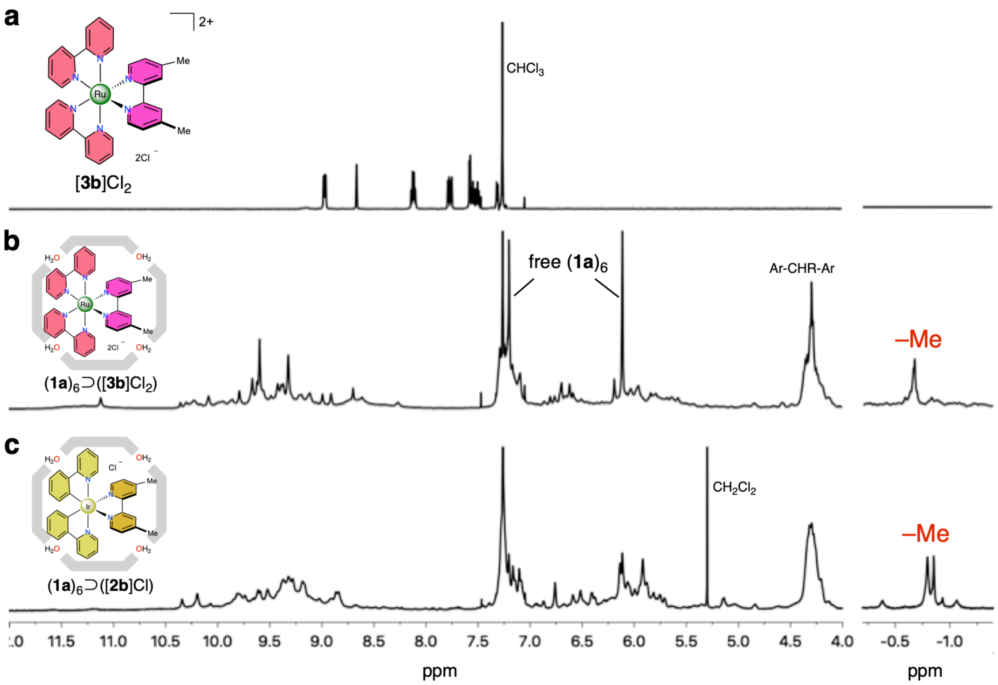

Supplementary Fig. 29 ^{1}H NMR spectra (500 MHz, $CDCl_3$, r.t.) of **a** [**3b**]Cl_2, **b** a mixture of (**1a**)$_6$ and [**3b**]Cl_2 after heating at 50 ºC for 1 h, and **c** host–guest complex (**1a**)$_6$⊃([**2b**]Cl). The signals were broadened and shifted to up-field region after host–guest formation, indicating that the Ru complex salt [**3b**]Cl_2 was encapsulated within the hexameric capsule.

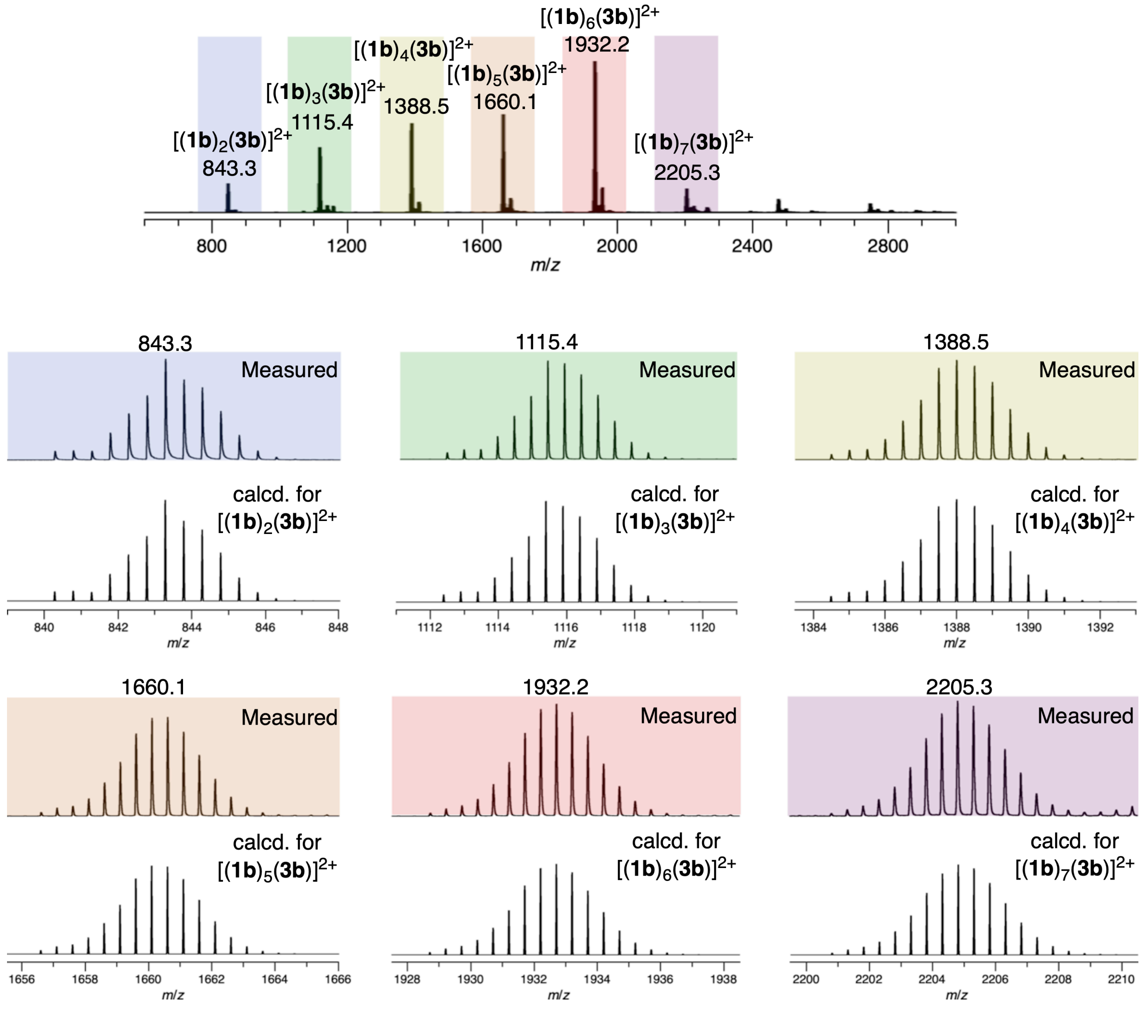

Supplementary Fig. 30 ESI-MS spectra of a mixture of resorcin[4]arene **1b** (R^1 = Me) and Ru complex salt [**3b**]Cl_2 in $CHCl_3$/acetone (2:1, v/v). The effective template effect from the complex cation affords supramolecular Ru complexes encapsulated by resorcin[4]arene oligomers in the gas-phase.

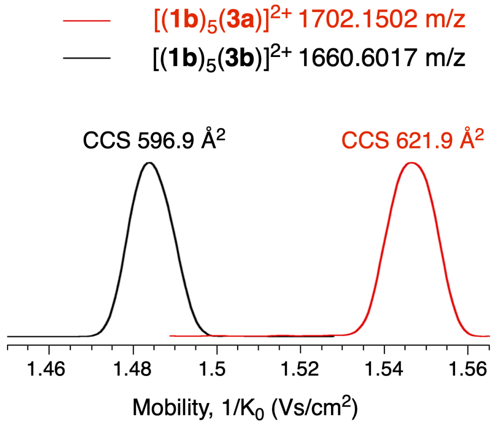

Supplementary Fig. 31 Ion Mobility spectra of [(**1b**)$_5$(**3a**)]$^{2+}$ and [(**1b**)$_5$(**3b**)]$^{2+}$. The collisional cross-section (CCS) of [(**1b**)$_5$(**3a**)]$^{2+}$ is significantly larger than that of [(**1b**)$_5$(**3b**)]$^{2+}$, indicating that the sterically demanding ^{t}Bu groups in [**3a**]$^{2+}$ promote to form a larger structure than templated by [**3b**]$^{2+}$.

X-ray structural determination

Single crystals of (**1c**)$_3$⊃[**3a**]Cl_2 suitable for X-ray crystallographic analysis were obtained as red platelet crystals from the MeOH-H_2O mixed solution (2:1, v/v) of resorcin[4]arene **1c** and ruthenium complex [Ru(bpy)$_2$(^{t}Bu$_2$bpy)]Cl_2 ([**3a**]Cl_2). As the single crystals were highly fragile owing to solvent loss, coating a crystal with a protectant (FLUOROLUBE® oil) and mounting it onto X-ray diffractometer (Rigaku Saturn724) were needed to be manipulated quickly. X-ray diffraction data were collected at 93 K. The crystal structure was initially solved by direct method (SHELXS-97). The positional and thermal parameters of non-H atoms were refined anisotropically by the full-matrix least-squares method except for disordering moieties. All calculations were performed using the CrystalStructure crystallographic software package except for refinement, which was performed using SHELXL-2014/7.

Crystal data for (**1c**)$_3$⊃[**3a**]Cl_2: $C_{172}H_{214}Cl_2N_6O_{43}Ru$, M = 322.57, space group P–1 (no. 2), a = 15.793(2), b = 20.409(3), c = 30.746(5) Å, α = 85.012(5)°, β = 83.649(6)°, γ = 78.882(5)°, V = 9642.6(2) Å^3, Z = 2, F(000) = 3420, Dc = 1.111 g/cm^3, μ(MoKα) = 1.79 cm^{-1}, T = 93(3), 2043 variables refined with 42538 reflections with $I > 2\sigma(I)$ to $R1$ = 0.1649. High flexibility of the molecules and the large solvent area in the crystal lattice required stereochemical restraints to stabilize the model structure. A combination of some restraints (SIMU and DELU) was applied for the refinement of anisotropic displacement parameters (ADP) for the molecules. The solvent molecules (MeOH and H_2O) were assigned for the residual electron density in the large solvent area, that were refined isotropically. The contribution of scattered electron density from severely disordered solvent molecules at the center of the solvent area, which could not be modeled reasonably, were handled using the SQUEEZE routine in PLATON program. This crystal structure contains several checkCIF alerts (Alert A and B), owing to the highly flexible framework and the disordered solvent molecules. The alerts and responses are given below.

PLAT084_ALERT_3_A
PROBLEM: High wR2 Value (i.e. > 0.25) 0.48
PLAT082_ALERT_2_B
PROBLEM: High R1 Value 0.16
PLAT213_ALERT_2_B
PROBLEM: Atom O63 has ADP max/min Ratio 5.0
PLAT250_ALERT_2_B
PROBLEM: Large U3/U1 Ratio for Average U(i, j) Tensor 5.2
RESPONSE: These alerts originated from the high flexibility of the structure and severely disordered solvent molecules in the large solvent area. The molecules are weakly trapped in the crystal, giving the large thermal ellipsoids. Therefore, this refinement had extensively used restraints in a macromolecular fashion to stabilize the flexible moieties. The geometry and ADP restraints ensure a chemically and physically meaningful structural model.

PLAT260_ALERT_2_B
PROBLEM: Large Average Ueq of Residue Including O2S 0.304
PLAT306_ALERT_2_B
PROBLEM: Isolated Oxygen Atom (H–atoms Missing ?) O3S
PROBLEM: Isolated Oxygen Atom (H–atoms Missing ?) O4S

PROBLEM: Isolated Oxygen Atom (H–atoms Missing ?) O5S
PROBLEM: Isolated Oxygen Atom (H–atoms Missing ?) O6S
PROBLEM: Isolated Oxygen Atom (H–atoms Missing ?) O7S
PROBLEM: Isolated Oxygen Atom (H–atoms Missing ?) O8S
PROBLEM: Isolated Oxygen Atom (H–atoms Missing ?) O9S
PROBLEM: Isolated Oxygen Atom (H–atoms Missing ?) O10S
PROBLEM: Isolated Oxygen Atom (H–atoms Missing ?) O11S
PROBLEM: Isolated Oxygen Atom (H–atoms Missing ?) O12S
PROBLEM: Isolated Oxygen Atom (H–atoms Missing ?) O13S
PROBLEM: Isolated Oxygen Atom (H–atoms Missing ?) O14S

PLAT430_ALERT_2_B
PROBLEM: Short Inter D...A Contact O1S ..O8S · 2.73 Ang.
PROBLEM: Short Inter D...A Contact O2S ..O9S · 2.74 Ang.
PROBLEM: Short Inter D...A Contact O3S ..O4S · 2.80 Ang.
PROBLEM: Short Inter D...A Contact O4S ..O8S · 2.71 Ang.
PROBLEM: Short Inter D...A Contact O6S ..O9S · 2.79 Ang.
PROBLEM: Short Inter D...A Contact O8S ..O9S · 2.75 Ang.

RESPONSE: These alerts derived from handling the disordered solvent molecules (O1S to O14S). This crystal contains large solvent area, where solvent molecules (MeOH and H_2O) are disordered. The solvent molecules were refined isotropically on a basis of scattered electron density in the solvent area (Supplementary Fig. 35). Owing to the less ordered hydrogen-bonding network of the OH moieties, the position of hydrogen atoms could not be defined.

Metrical parameters for the crystal structure of (**1c**)$_3$⊃[**3**]Cl_2, are available free of charge from the Cambridge Crystallographic Data Centre under reference numbers CCDC-2152087.

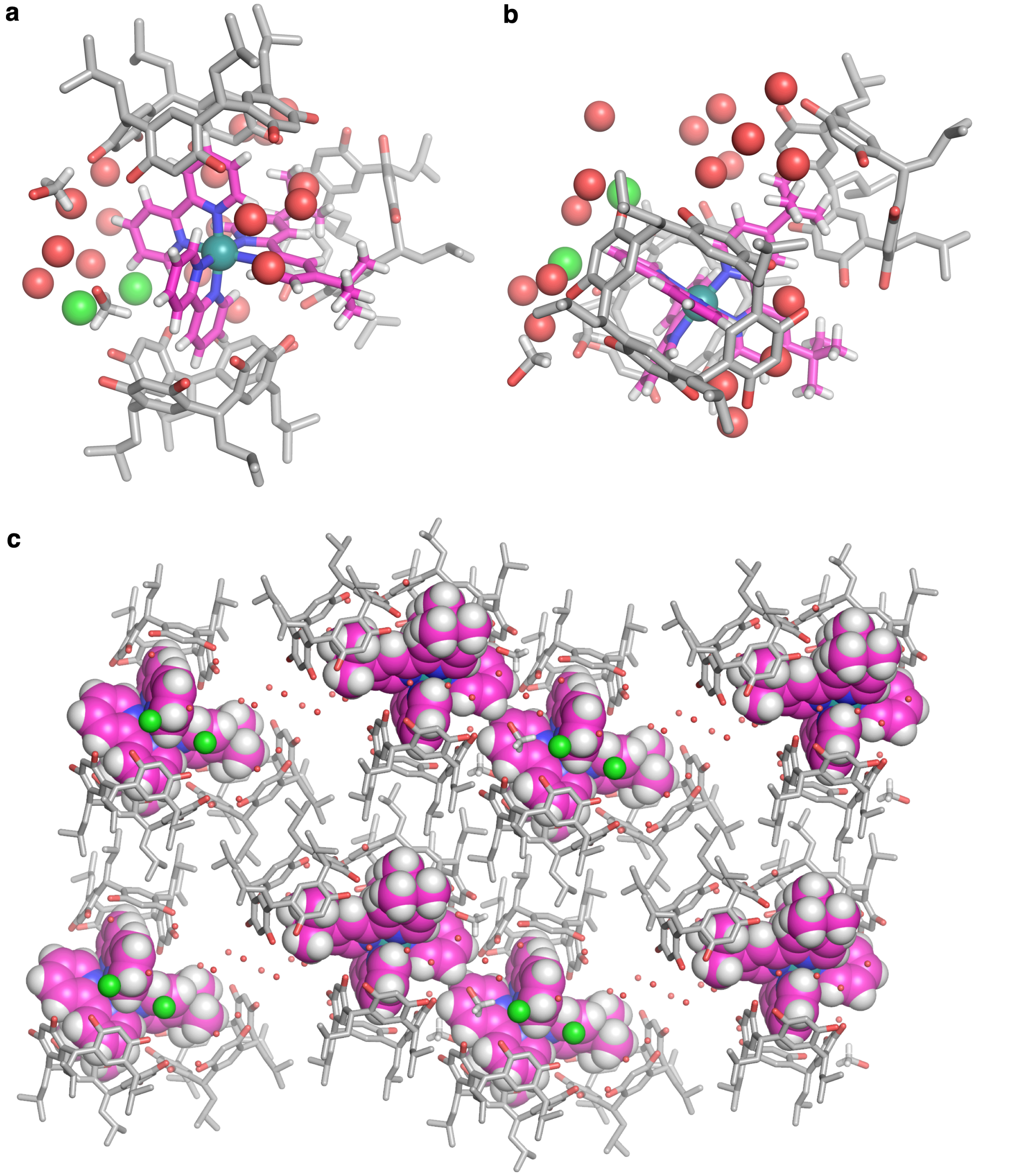

Supplementary Fig. 32 Single crystal X-ray structure of inclusion complex $(\mathbf{1c})_3\supset[\mathbf{3a}]\mathrm{Cl}_2$ (Ru, turquoise blue; Cl, green; O, red; N, blue; C, grey and pink; H, sky grey); **a** front view, and **b** top view. Disordered ^{i}Bu groups in **1c** and hydrogen atoms are omitted for clarity. The Ru complex is non-covalently sandwiched by two resorcin[4]arene units by π–π interactions between electron-deficient pyridine moieties in $[\mathbf{3a}]^{2+}$ and electron-rich aromatic surfaces of **1c**, and one of the ^{t}Bu groups in $[\mathbf{3a}]^{2+}$ is recognized by the third resorcin[4]arene via multiple CH···π interactions. This partial host–guest motif nicely supports the solution structure proposed on the basis of the NMR experiments. The chloride ions of $[\mathbf{3a}]\mathrm{Cl}_2$ are trapped by hydrogen-bonds from OH groups of **1c** to assist ion-pair formation upon encapsulation, being also in good agreement with the proposed anion-mediated host–guest formation mechanism in solution.

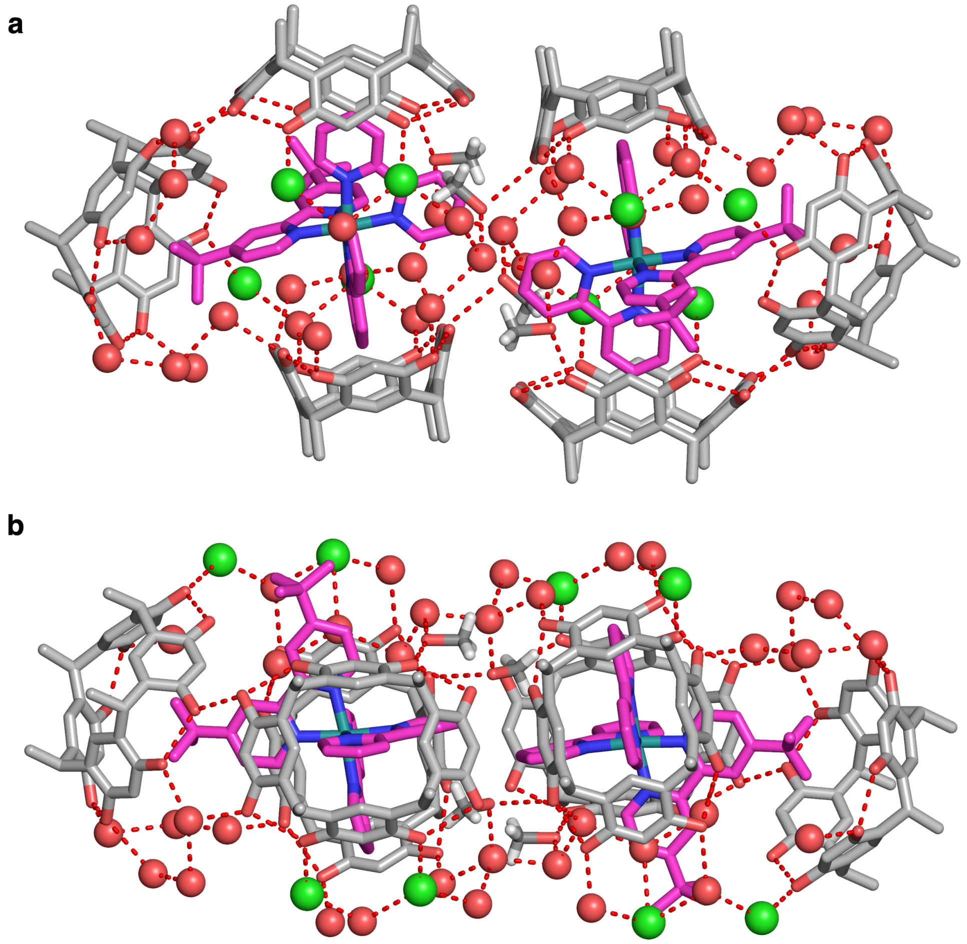

Supplementary Fig. 33 Hydrogen-bonding network of inclusion complex $(\mathbf{1c})_3\supset[\mathbf{3a}]\mathrm{Cl}_2$ (Ru, turquoise blue; Cl, green; O, red; N, blue; C, grey and pink); **a** front view, and **b** top view. The ^{t}Bu groups of **1c** and hydrogen atoms are omitted for clarity. The resorcin[4]arene trimer was stabilized by the host–guest interactions and connected via multiple hydrogen-bonds among the OH groups of **1c**, solvent molecules, and Cl anions.

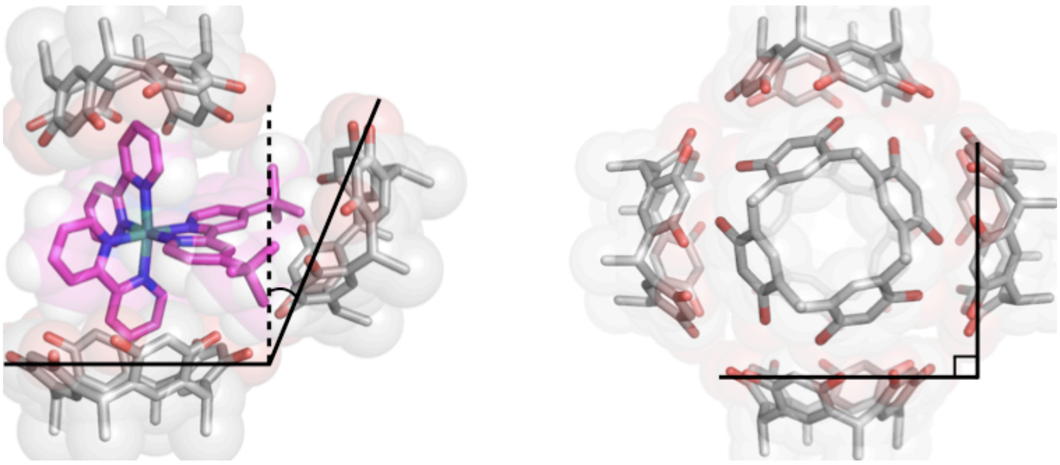

Supplementary Fig. 34 Orientation of resorcin[4]arene units in $(\mathbf{1c})_3\supset[\mathbf{3a}]\mathrm{Cl}_2$ and the hexameric capsule. The resorcin[4]arene units are diagonally oriented in $(\mathbf{1c})_3\supset[\mathbf{3a}]\mathrm{Cl}_2$ owing to steric effect from ^{t}Bu groups in $[\mathbf{3a}]^{2+}$. This X-ray structure suggests that the ^{t}Bu group effectively destabilizes the hexameric capsule structure to promote the formation of the distorted hexameric resorcin[4]arene capsule.

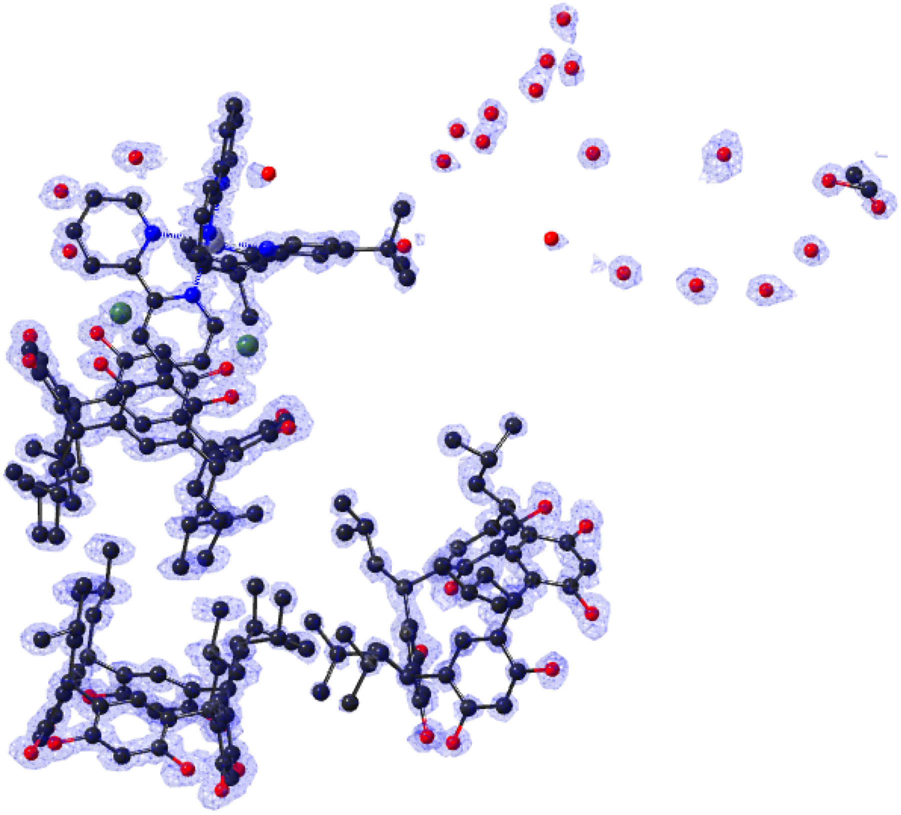

Supplementary Fig.35 Electron density map of (**1c**)$_3$⊃[**3a**]Cl_2. Blue mesh represents observed electron density at 1.0 e/Å^3 level. The solvent molecules were refined on a basis of the scattered electron density in the solvent area.

Photophysical studies

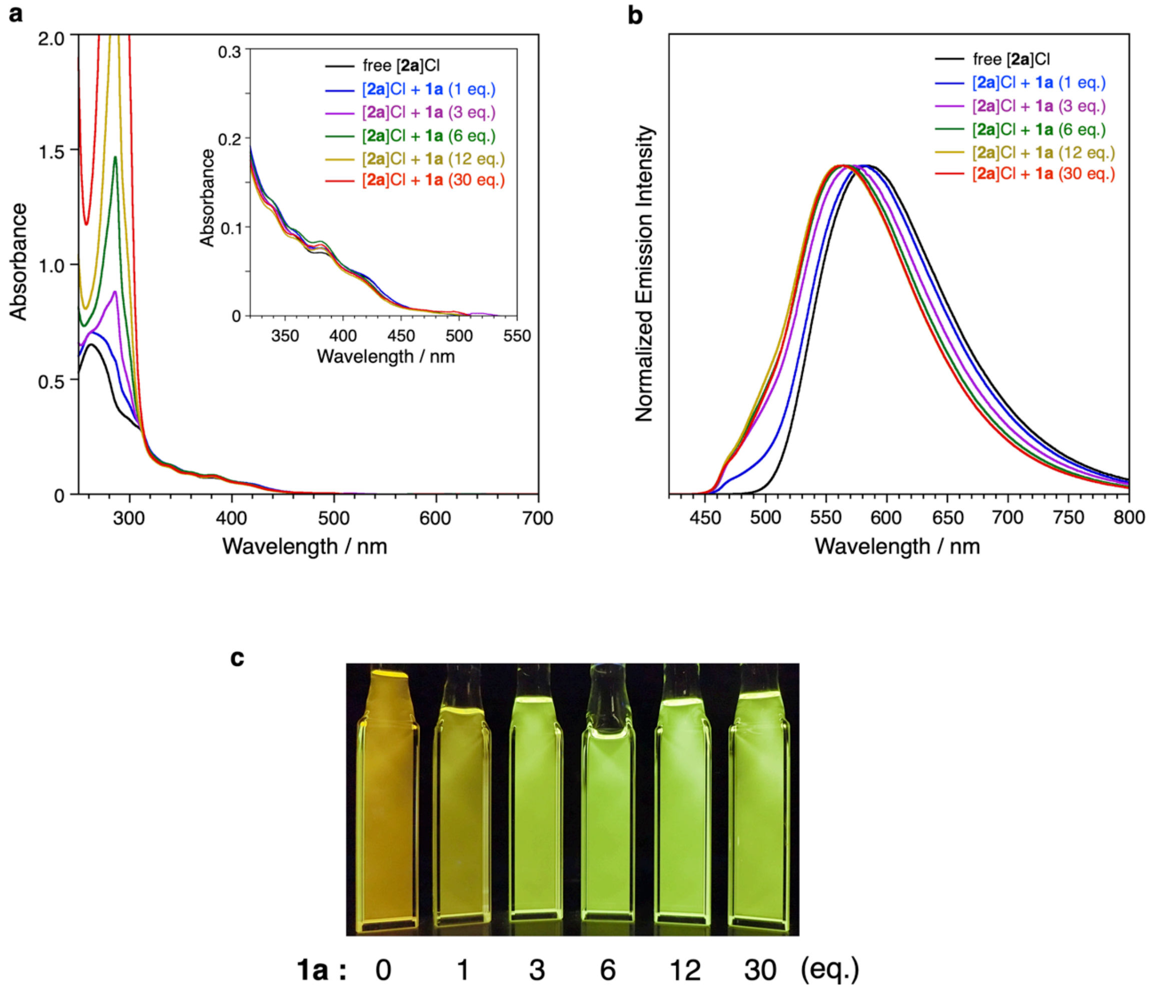

Supplementary Fig. 36 a UV-vis absorption and **b** normalized emission spectra of the [**2a**]Cl in the presence of **1a** (λ_{ex} = 400 nm), and **c** photographic images of the $CHCl_3$ solutions (10 μM for [**2a**]Cl) with various amounts of **1a** under photoirradiation (λ_{ex} = 365 nm). The samples were heated at 50 ºC for 1 min before the measurements. The absorption spectra of [**2a**]Cl did not change significantly after encapsulation, meanwhile the emission spectra of $[\mathbf{2a}]^+$ showed blue-shift upon addition of **1a**.

Supplementary Table 1. Photophysical data (10 μM for [**2a**]Cl, $CHCl_3$, r.t.) with **1a**.

[**1a**] [μM]	λ_{em} [nm]	Φ [%]	A_1	τ_1 [ns]	A_2	τ_2 [ns]	τ_{ave} [ns]	k_r [$\times 10^5$ s^{-1}]	k_{nr} [$\times 10^5$ s^{-1}]
0	583	40	0.25^a	510^a			510^a	7.8	11.8
10	580	43	0.86	440	0.14	1300	720	6.0	7.9
30	573	55	0.71	610	0.29	1300	930	5.9	4.8
60	564	63	0.79	800	0.21	1470	1020	6.2	3.6
120	563	69	0.80	850	0.20	1540	1060	6.5	2.9
300	562	73	0.90	930	0.10	1800	1090	6.7	2.5

^{a} analysed by a single exponential decay.

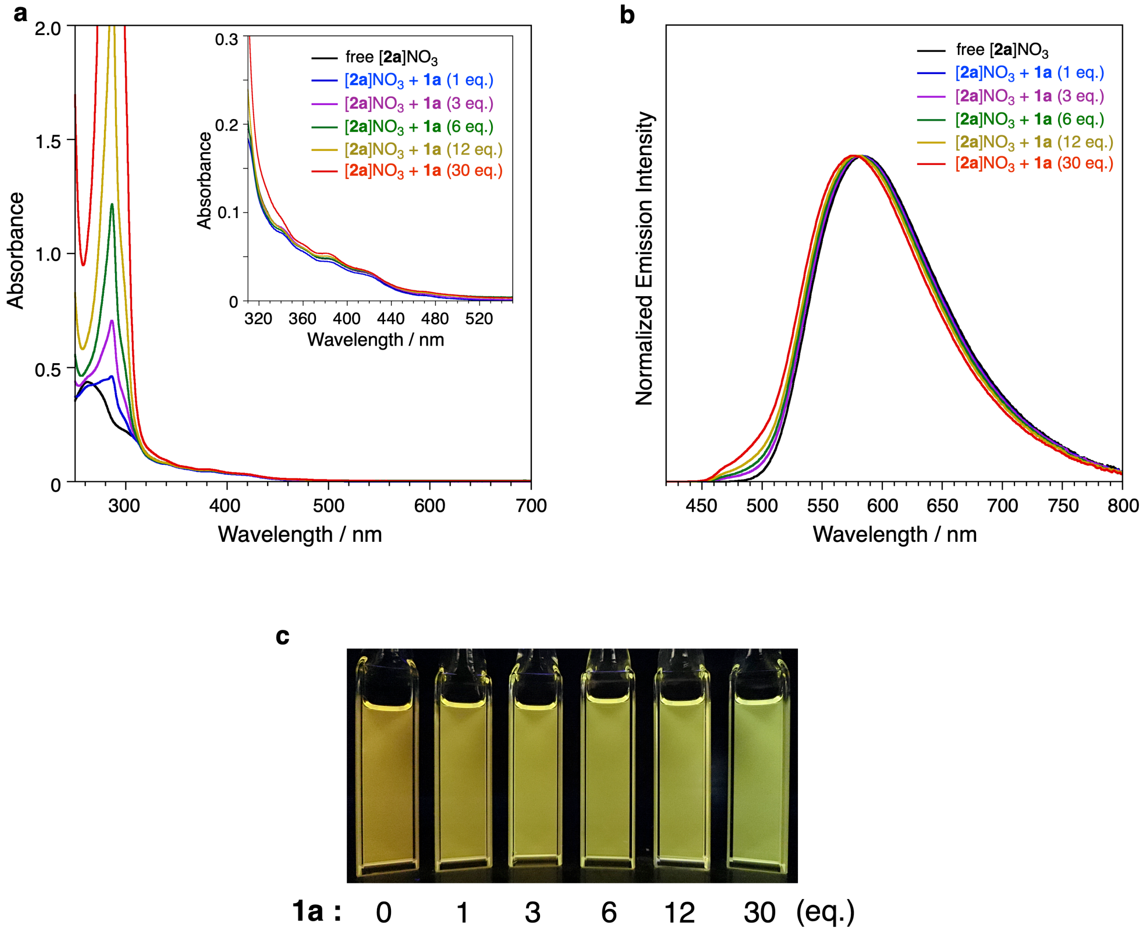

Supplementary Fig. 37 **a** UV-vis absorption and **b** normalized emission spectra of the [**2a**]NO_3 in the presence of **1a** (λ_{ex} = 400 nm), and **c** photographic images of the $CHCl_3$ solutions (10 μM for [**2a**]NO_3) with various amounts of **1a** under photoirradiation (λ_{ex} = 365 nm). The absorption spectra of [**2a**]NO_3 did not change significantly after encapsulation, meanwhile the emission spectra of [**2a**]$^+$ showed blue-shift upon addition of **1a**.

Supplementary Table 2. Photophysical data (10 μM for [**2a**]NO_3, $CHCl_3$, r.t.) with **1a**.

[**1a**] [μM]	λ_{em} [nm]	Φ [%]	A_1	τ_1 [ns]	A_2	τ_2 [ns]	τ_{ave} [ns]	k_r [$\times10^5$ s^{-1}]	k_{nr} [$\times10^5$ s^{-1}]
0	583	46	1.07[a]	580[a]			580[a]	7.9	9.3
10	581	43	0.90	560	0.10	1050	640	6.7	8.8
30	581	43	0.85	550	0.15	1040	670	6.4	8.5
60	580	43	0.80	510	0.20	1080	710	6.1	8.1
120	579	45	0.07	490	0.30	1030	750	6.0	7.4
300	577	50	0.59	610	0.41	1120	900	5.6	5.6

[a] analysed by a single exponential decay.

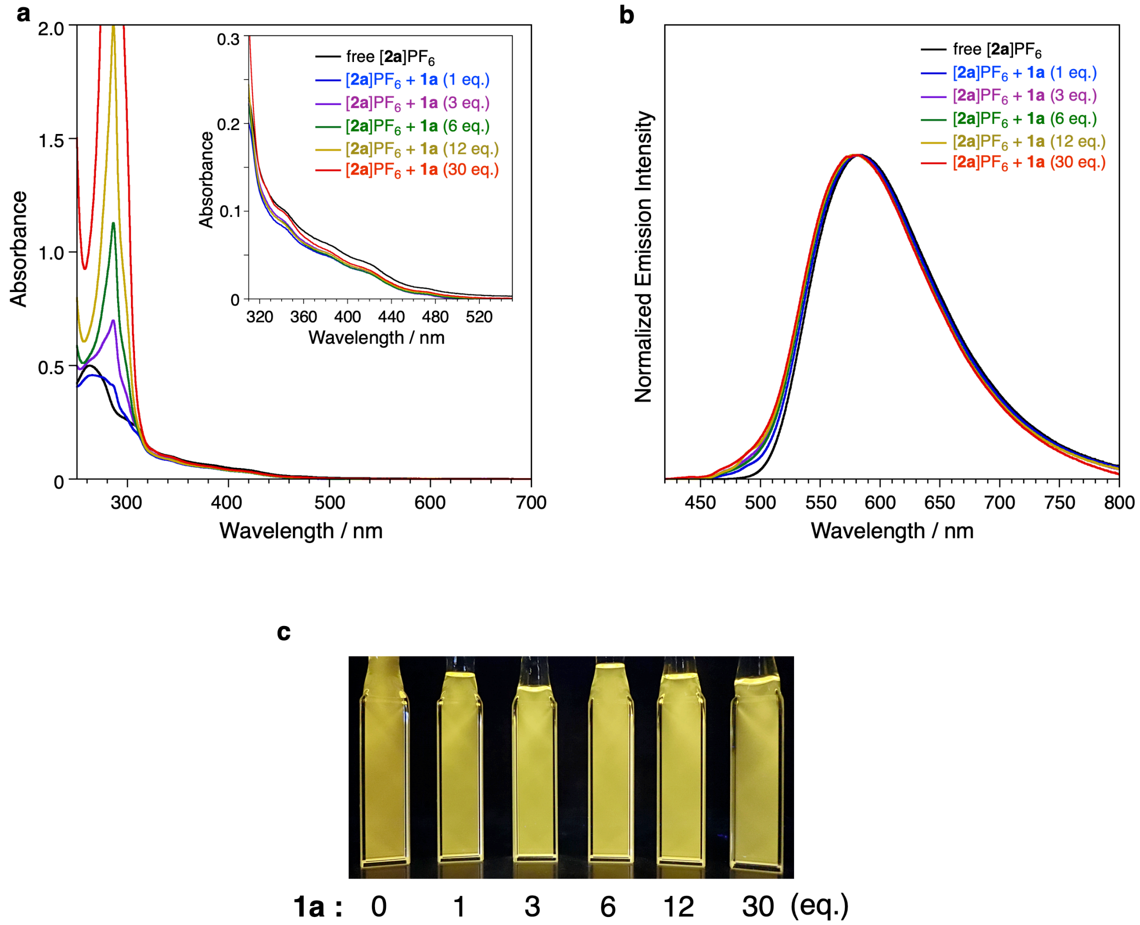

Supplementary Fig. 38 a UV-vis absorption and **b** normalized emission spectra of the [**2a**]PF_6 in the presence of **1a** (λ_{ex} = 400 nm), and **c** photographic images of the $CHCl_3$ solutions (10 μM for [**2a**]PF_6) with various amounts of **1a** under photoirradiation (λ_{ex} = 365 nm). Both the absorption and the emission spectra of [**2a**]PF_6 did not change significantly depending on the concentration of **1a** because the Ir complex salt with large counter anion was not adapted with the cavity of **1a**.

Supplementary Table 3. Photophysical data (10 μM for [**2a**]PF_6, $CHCl_3$, r.t.) with **1a**.

[**1a**] [μM]	λ_{em} [nm]	Φ [%]	A_1	τ_1 [ns]	A_2	τ_2 [ns]	τ_{ave} [ns]	k_r [$\times 10^5$ s^{-1}]	k_{nr} [$\times 10^5$ s^{-1}]
0	583	39	0.18^a	660^a			510^a	5.9	9.2
10	581	45	0.99	660	0.01	2010	700	6.4	7.8
30	581	49	0.98	740	0.02	2040	810	6.0	6.3
60	580	52	0.99	770	0.01	2200	830	6.3	5.8
120	580	53	0.97	790	0.03	2210	890	6.0	5.3
300	580	53	0.97	790	0.03	2230	900	5.9	5.2

^{a} analysed by a single exponential decay.

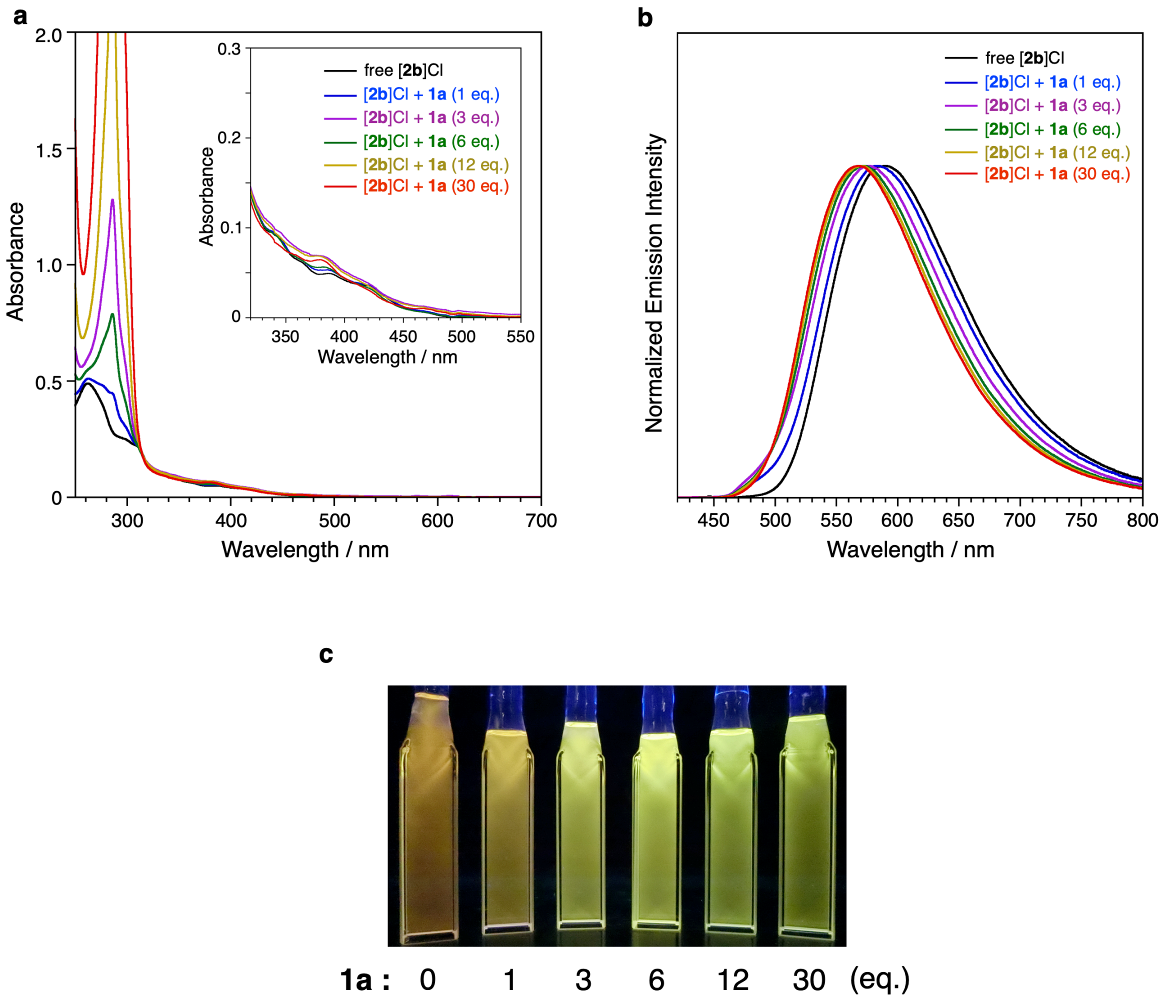

Supplementary Fig. 39 **a** UV-vis absorption and **b** normalized emission spectra of the [**2b**]Cl in the presence of **1a** (λ_{ex} = 400 nm), and **c** photographic images of the $CHCl_3$ solutions (10 μM for [**2b**]Cl) with various amounts of **1a** under photoirradiation (λ_{ex} = 365 nm). The samples were heated at 50 ºC for 1 min before the measurements. The absorption spectra of [**2b**]Cl did not change significantly after encapsulation, meanwhile the emission spectra of $[\mathbf{2b}]^+$ showed blue-shift upon addition of **1a**.

Supplementary Table 4. Photophysical data (10 μM for [**2b**]Cl, $CHCl_3$, r.t.) with **1a**.

[**1a**] [μM]	λ_{em} [nm]	Φ [%]	A_1	τ_1 [ns]	A_2	τ_2 [ns]	τ_{ave} [ns]	k_r [$\times 10^5$ s^{-1}]	k_{nr} [$\times 10^5$ s^{-1}]
0	590	26	0.20^a	450^a			450^a	5.8	16.4
10	581	32	0.84	470	0.16	1140	680	4.7	10.0
30	577	37	0.78	500	0.22	1110	740	5.0	8.5
60	572	39	0.62	500	0.38	1090	840	4.6	7.3
120	570	42	0.58	520	0.42	1200	950	4.4	6.1
300	568	47	0.51	540	0.49	1190	980	4.8	5.4

^{a} analysed by a single exponential decay.

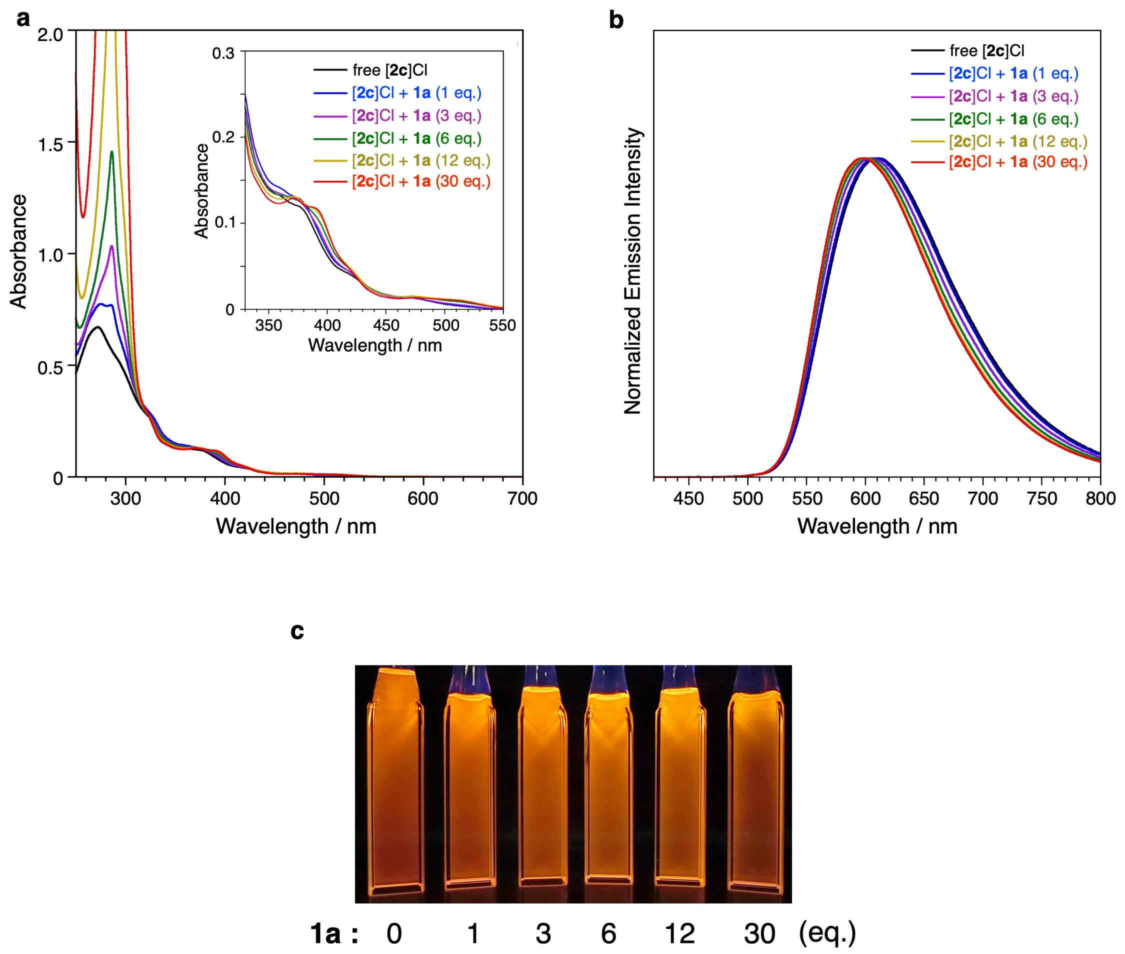

Supplementary Fig. 40 **a** UV-vis absorption and **b** normalized emission spectra of the [**2c**]Cl in the presence of **1a** (λ_{ex} = 400 nm), and **c** photographic images of the $CHCl_3$ solutions (10 μM for [**2c**]Cl) with various amounts of **1a** under photoirradiation (λ_{ex} = 365 nm). The samples were heated at 50 ºC for 1 min before the measurements. Both the absorption and the emission spectra of [**2c**]Cl did not change significantly depending on the concentration of **1a**, because the Ir complex cation was not adapted with the capsule (**1a**)$_n$.

Supplementary Table 5. Photophysical data (10 μM for [**2c**]Cl, $CHCl_3$, r.t.) with **1a**.

[**1a**] [μM]	λ_{em} [nm]	Φ [%]	A_1	τ_1 [ns]	A_2	τ_2 [ns]	τ_{ave} [ns]	k_r [$\times10^5$ s^{-1}]	k_{nr} [$\times10^5$ s^{-1}]
0	611	35	0.18[a]	510[a]			510[a]	6.9	12.7
10	610	36	0.79	420	0.21	700	510	7.1	12.5
30	605	38	0.84	460	0.16	750	530	7.1	11.7
60	601	44	0.91	500	0.09	840	550	8.0	10.2
120	599	48	0.95	560	0.05	960	590	8.1	8.8
300	598	50	0.98	620	0.02	1260	650	7.7	7.7

[a] analysed by a single exponential decay.

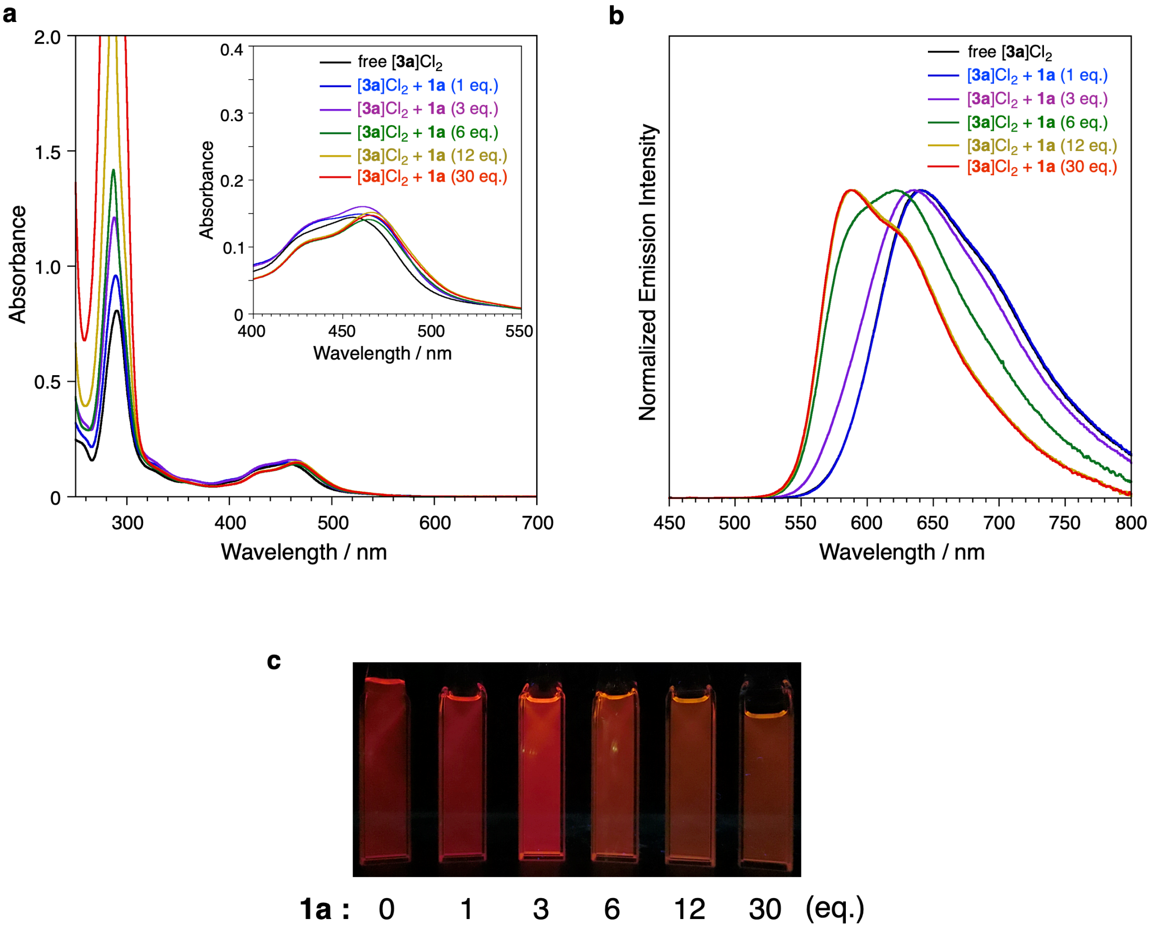

Supplementary Fig. 41 **a** UV-vis absorption and **b** normalized emission spectra of the [**3a**]Cl_2 in the presence of **1a** (λ_{ex} = 400 nm), and **c** photographic images of the $CHCl_3$ solutions (10 μM for [**3a**]Cl_2) with various amounts of **1a** under photoirradiation (λ_{ex} = 365 nm). The samples were heated at 50 °C for 1 min before the measurements. The absorption spectra of [**3a**]Cl_2 did not change significantly after encapsulation, meanwhile the emission spectra of [**3a**]$^{2+}$ showed blue-shift upon addition of **1a**.

Supplementary Table 6. Photophysical data (10 μM for [**3a**]Cl_2, $CHCl_3$, r.t.) with **1a**.

[**1a**] [μM]	λ_{em} [nm]	Φ [%]	A_1	τ_1 [ns]	A_2	τ_2 [ns]	τ_{ave} [ns]	k_r [$\times10^5$ s^{-1}]	k_{nr} [$\times10^5$ s^{-1}]
0	641	12	0.18[a]	610[a]			610[a]	2.0	14.4
10	641	11	0.27	450	0.73	650	610	1.8	14.6
30	636	10	0.82	440	0.18	1210	730	1.4	12.3
60	622	7	0.59	300	0.41	1390	1140	6.1	8.2
120	589	6	0.58	260	0.42	1530	1290	4.6	7.3
300	587	5	0.60	240	0.40	1540	1300	3.9	7.4

[a] analysed by a single exponential decay.

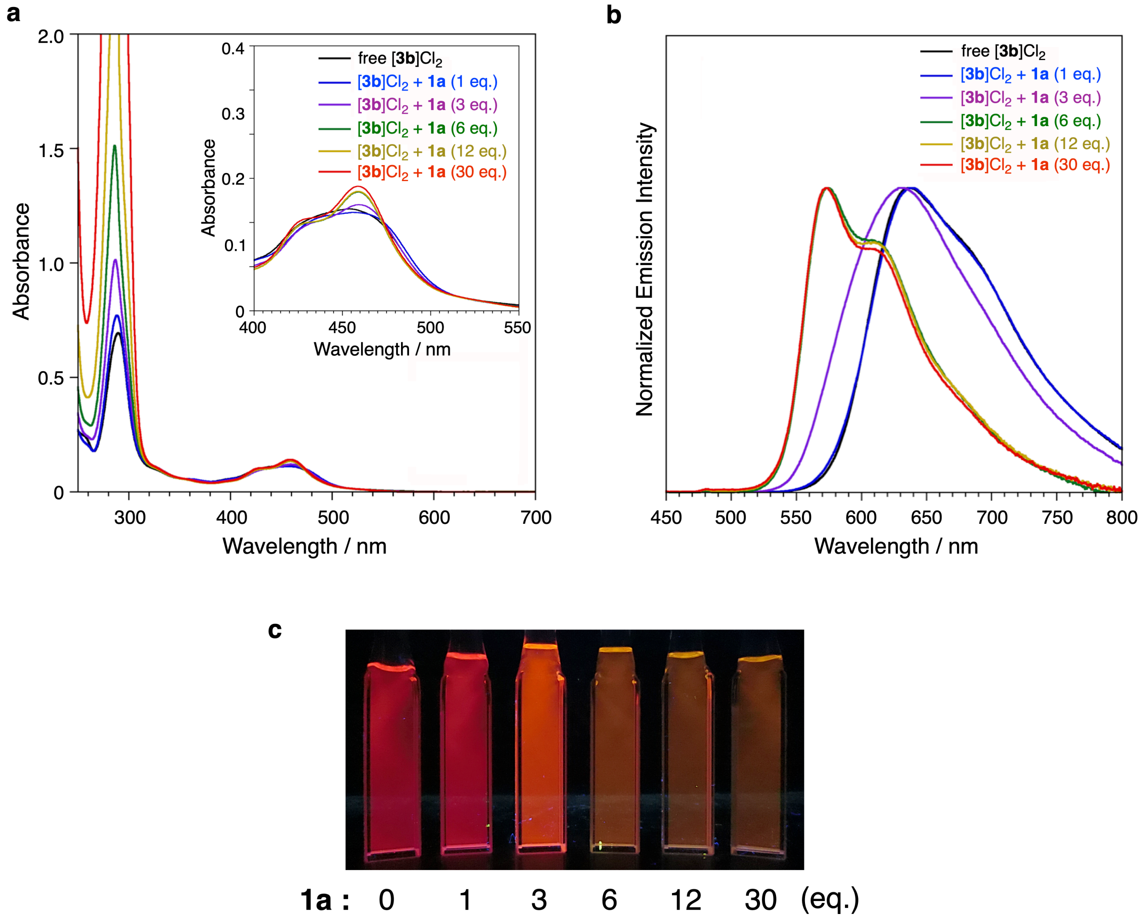

Supplementary Fig. 42 **a** UV-vis absorption and **b** normalized emission spectra of the [**3b**]Cl_2 in the presence of **1a** (λ_{ex} = 400 nm), and **c** photographic images of the $CHCl_3$ solutions (10 μM for [**3b**]Cl_2) with various amounts of **1a** under photoirradiation (λ_{ex} = 365 nm). The absorption spectra of [**3b**]Cl_2 did not change significantly after encapsulation, meanwhile the emission spectra of [**3b**]$^{2+}$ showed blue-shift upon addition of **1a**. [a] After encapsulation by heating at 50 °C for 1 min.

Supplementary Table 7. Photophysical data (10 μM for [**3b**]Cl_2, $CHCl_3$, r.t.) with **1a**.

[**1a**] [μM]	λ_{em} [nm]	Φ [%]	A_1	τ_1 [ns]	A_2	τ_2 [ns]	τ_{ave} [ns]	k_r [×10^5 s^{-1}]	k_{nr} [×10^5 s^{-1}]
0	638	14	0.006[a]	700[a]			700[a]	2.0	12.3
10	638	14	0.13	260	0.87	680	650	2.1	13.1
30	631	10	0.69	360	0.31	1000	730	1.4	12.3
60	573	4	0.91	180	0.09	1240	620	0.65	15.6
120	573	2	0.96	180	0.04	980	320	0.62	30.3
300	573	2	0.95	180	0.05	850	310	0.63	30.7

[a] analysed by a single exponential decay.

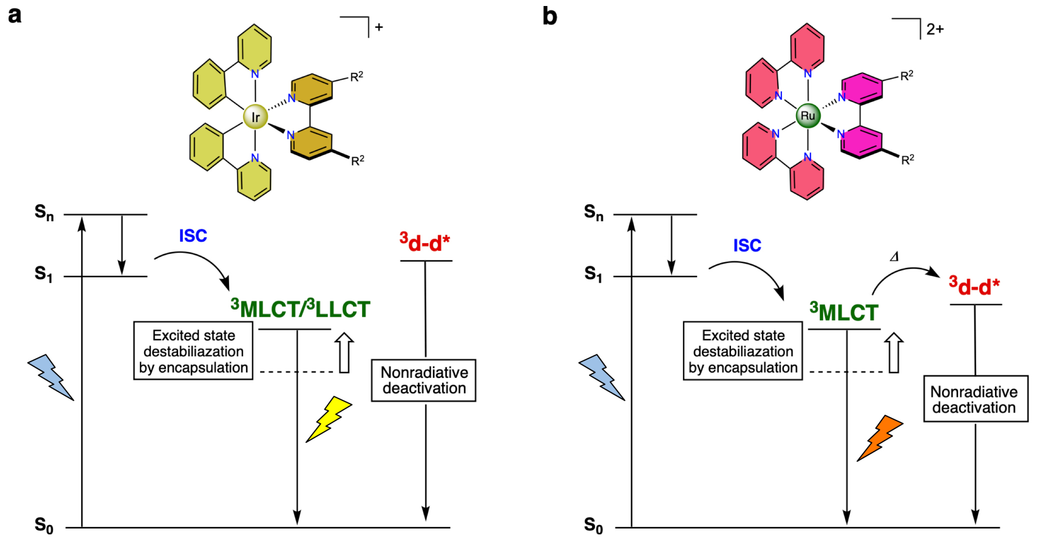

Supplementary Fig. 43 Proposed photo-excited dynamics of **a** the Ir complex cation, and **b** the Ru complex cation after host–guest formation. The Ir complex, whose triplet d-d* excited state lays at much higher level than the lowest ^{3}MLCT/^{3}LLCT excited state, can show EIEE behavior by encapsulation. On the other hand, as the energy level of the ^{3}MLCT state is destabilized by encapsulation and its energy level might be comparable with the d-d* excited state of the Ru complex,[6] thermal activation from ^{3}MLCT to the d-d* excited state is suggested to be promoted, causing reduced emission quantum yield and lifetime.

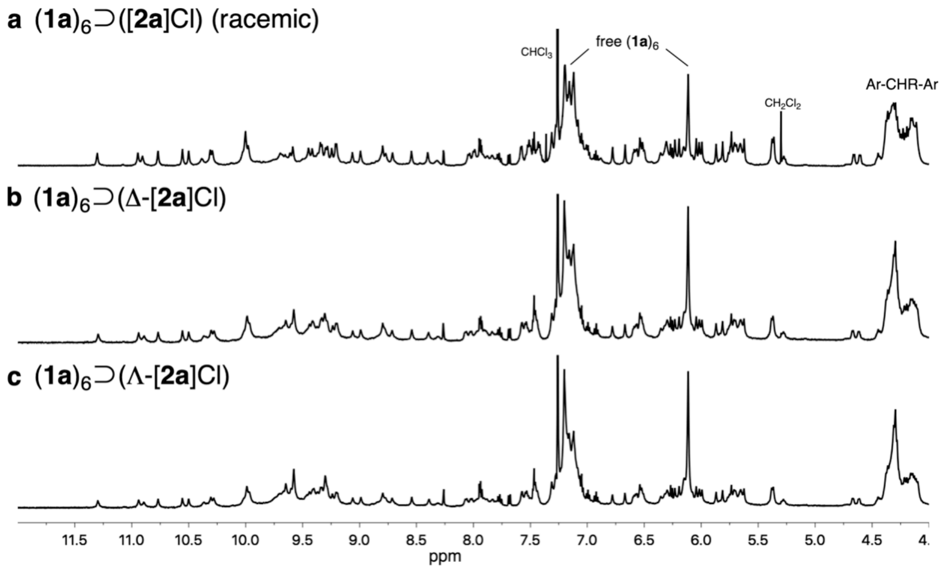

Supplementary Fig. 44 ^{1}H NMR spectra (500 MHz, $CDCl_3$, r.t.) of host–guest complex [(**1a**)$_6$⊃[**2a**]Cl] obtained from **a** racemic compound [**2a**]Cl, **b** Δ-[**2a**]Cl, and **c** Λ-[**2a**]Cl. These results clearly showed that the symmetry-breaking assembly was independent on the chirality of the guest.

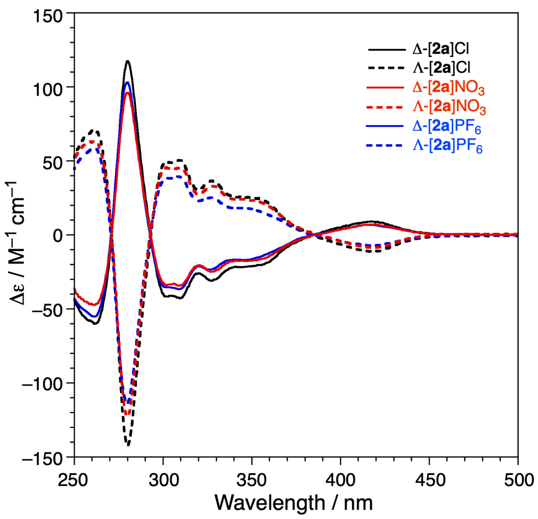

Supplementary Fig. 45 Circular dichroism (CD) spectra (10 μM, $CHCl_3$, r.t.) of enantiomerically pure Λ- and Δ- Ir complex salts, showing that the CD spectra of the complex cation are irrespective to their counter anions.

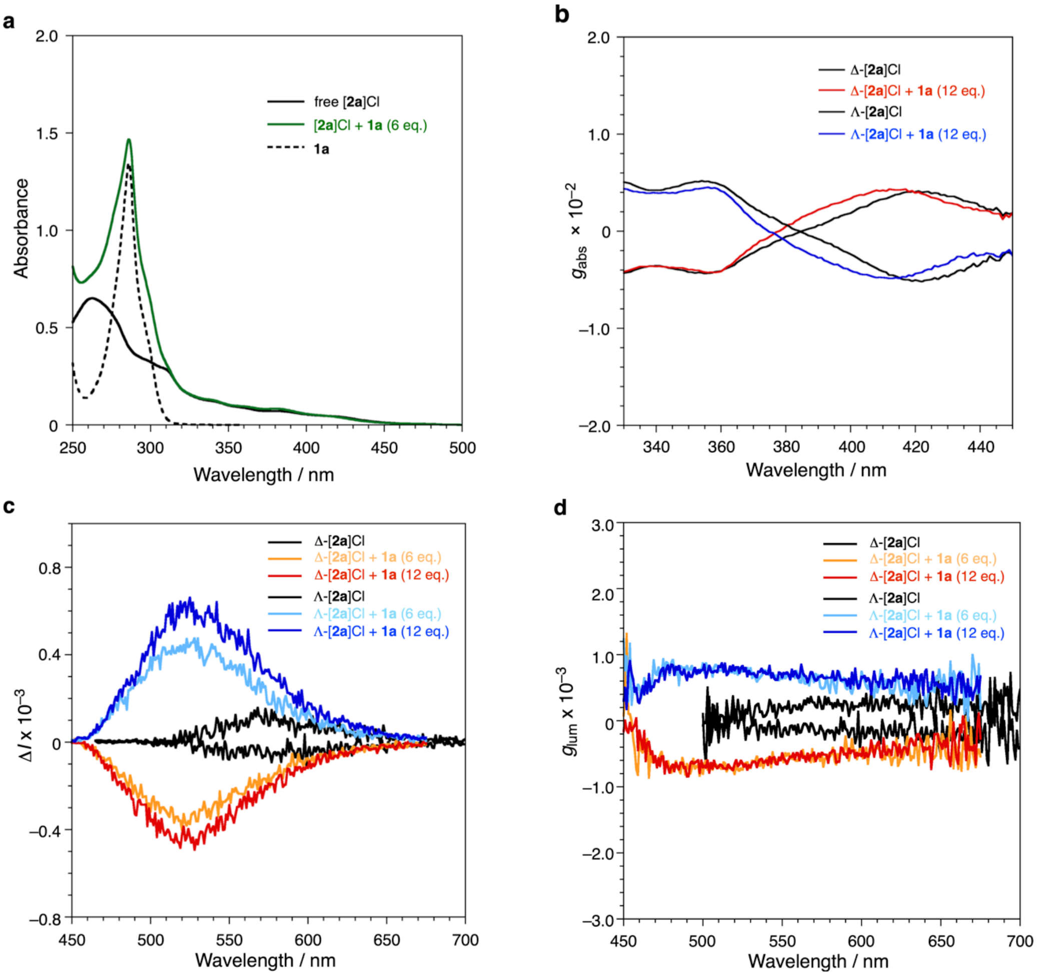

Supplementary Fig. 46 a UV-vis absorption spectra ($CHCl_3$, r.t.) of free [**2a**]Cl (10 μM) and **1a** (60 μM), **b** dissymmetry factor (g_{abs}) of the CD property, which was estimated by the equation ($g_{abs} = \Delta\varepsilon_{CD}/\ \varepsilon_{UV\text{-}Vis}$), **c** Circularly polarized luminescence (CPL) spectra (50 μM, $CHCl_3$, r.t.) of enantiomerically pure Ir complex [**2a**]Cl (50 μM) with resorcin[4]arene monomer **1a** (0, 300, 600 μM), and **d** dissymmetry factor (g_{lum}) of the CPL property. λ_{ex} = 350 nm. The samples were heated at 50 ºC for 1 min before the measurements.

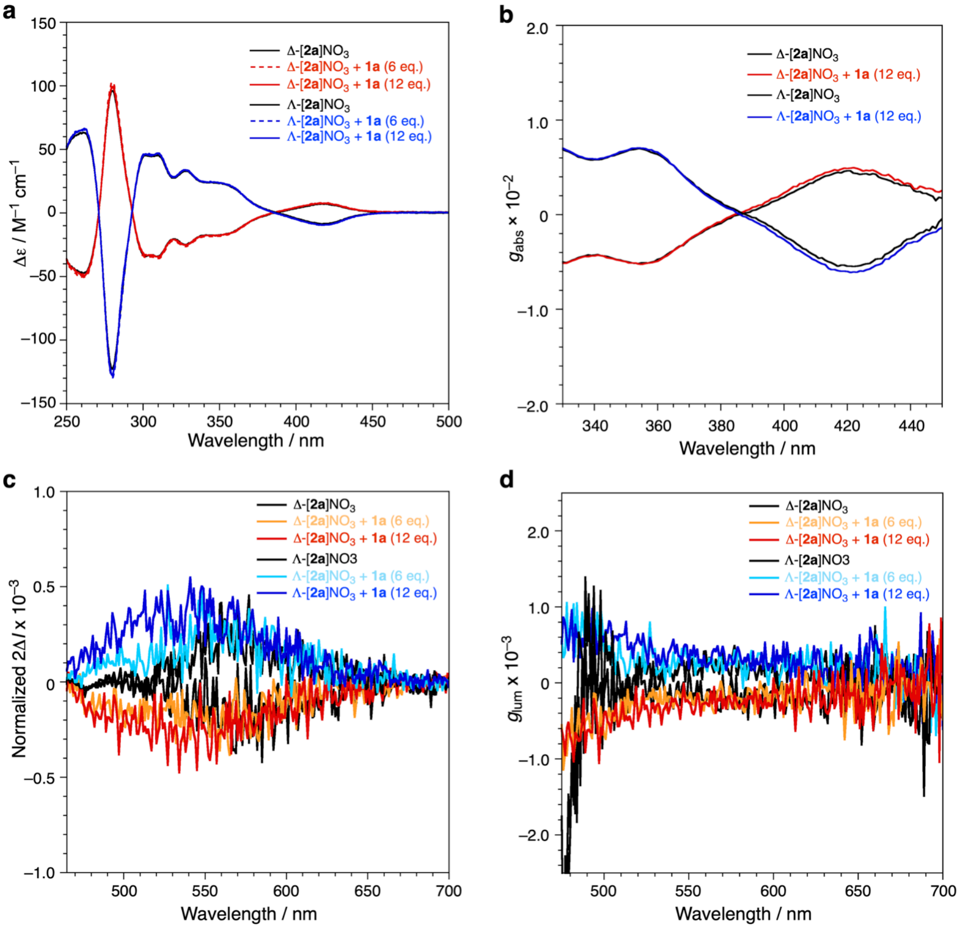

Supplementary Fig. 47 a Circular dichroism (CD) spectra (10 μM, $CHCl_3$, r.t.) of Δ- and Λ-[**2a**]NO_3 in the presence of resorcin[4]arene **1a** (0, 60, 120 μM), **b** dissymmetry factor (g_{abs}) of the CD property, which was estimated by the equation ($g_{abs} = \Delta\varepsilon_{CD}/\varepsilon_{UV\text{-}Vis}$), and **c** circularly polarized luminescence (CPL) spectra (50 μM, $CHCl_3$, r.t.) of enantiomerically pure Ir complex [**2a**]NO_3 (50 μM) with resorcin[4]arene monomer **1a** (0, 300, 600 μM) and **d** dissymmetry factor (g_{lum}) of the CPL property. λ_{ex} = 350 nm. The samples were heated at 50 ºC for 1 min before the measurements. These results clearly show that the CD and CPL spectra of the NO_3 salt are not altered significantly upon encapsulation by the symmetric hexameric capsule.

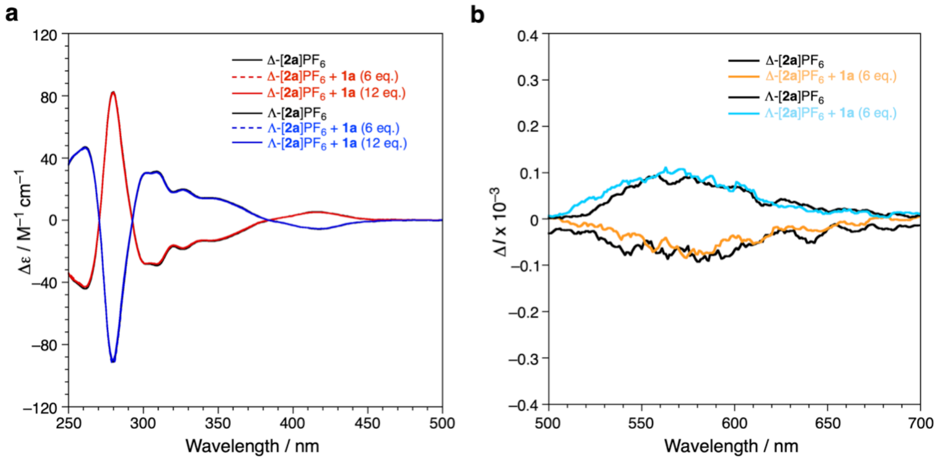

Supplementary Fig. 48 a Circular dichroism (CD) spectra (10 μM, $CHCl_3$, r.t.) of Δ- and Λ-[**2a**]PF_6 in the presence of resorcin[4]arene **1a** (0, 60, 120 μM), and **b** circularly polarized luminescence (CPL) spectra (50 μM, $CHCl_3$, r.t.) of enantiomerically pure Ir complex [**2a**]PF_6 (50 μM) with resorcin[4]arene monomer **1a** (0, 300 μM). The samples were heated at 50 ºC for 1 min before the measurements. These results clearly show that the CD and CPL spectra of the PF_6 salt are not altered by the existence of the hexameric capsule.

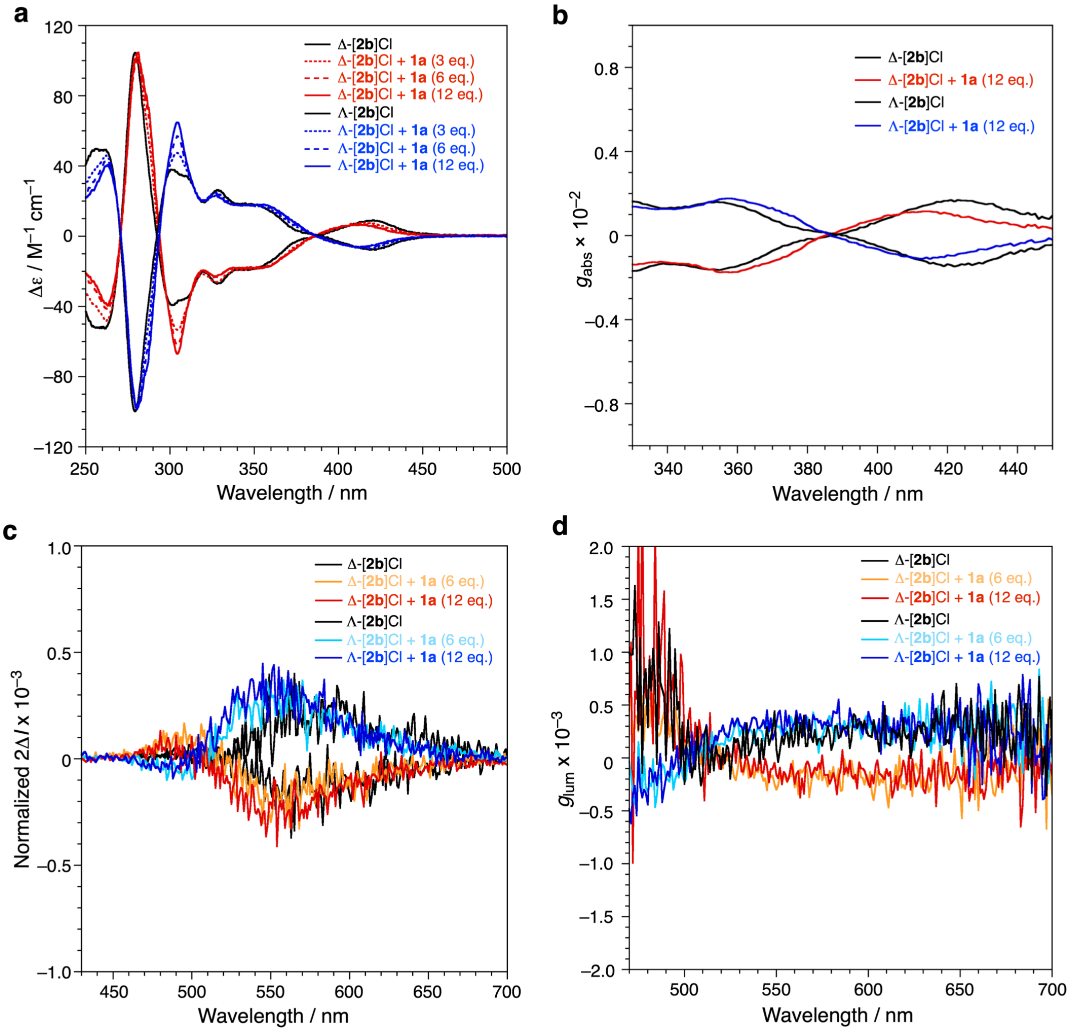

Supplementary Fig. 49 a Circular dichroism (CD) spectra (10 μM, $CHCl_3$, r.t.) of Δ- and Λ-[**2b**]Cl in the presence of resorcin[4]arene **1a** (0, 30, 60, 120 μM), **b** dissymmetry factor (g_{abs}) of the CD property, which was estimated by the equation ($g_{abs} = \Delta\varepsilon_{CD}/\varepsilon_{UV\text{-}Vis}$), **c** normalized circularly polarized luminescence (CPL) spectra (50 μM, $CHCl_3$, r.t.) of enantiomerically pure Ir complex [**2b**]Cl (50 μM) with resorcin[4]arene monomer **1a** (0, 300, 600 μM), which was obtained from division of the $2\Delta I$ curve by the maximum of the DC intensity, and **d** dissymmetry factor (g_{lum}) of the CPL property (λ_{ex} = 350 nm). The samples were heated at 50 ºC for 1 min before the measurements. In contrast to the results of [**2a**]Cl, Δ- and Λ-[**2b**]Cl did not show significant improvement of the dissymmetry factor (g_{lum}) upon addition of **1a**, probably because the small guest rotated freely within the typical resorcin[4]arene hexameric capsule and the C_2 molecular symmetry of the guest kept after encapsulation.

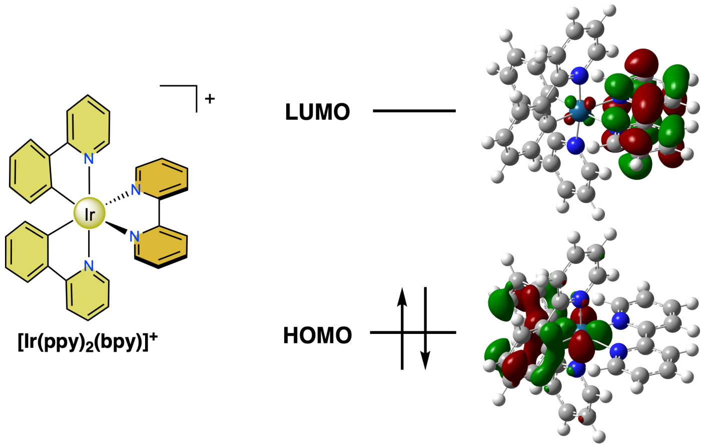

Supplementary Fig. 50 Frontier orbitals of Ir complex cation $[Ir(ppy)_2(bpy)]^+$ in the gas-phase, computed by the time-dependent density functional theory (TD-DFT) calculation using the B3LYP functional. In this calculation, for Ir atom, LANL2DZ basis set was used. For C, N, H atoms, cc-pVDZ basis sets were used. The photoluminescence of these kinds of Ir complex cations originates from ^{3}MLCT/^{3}LLCT mixed excited states. Orbital population on the two ppy ligands and two pyridine moieties on the bpy ligand are equivalent in a free complex cation, owing to the C_2 symmetric structure.

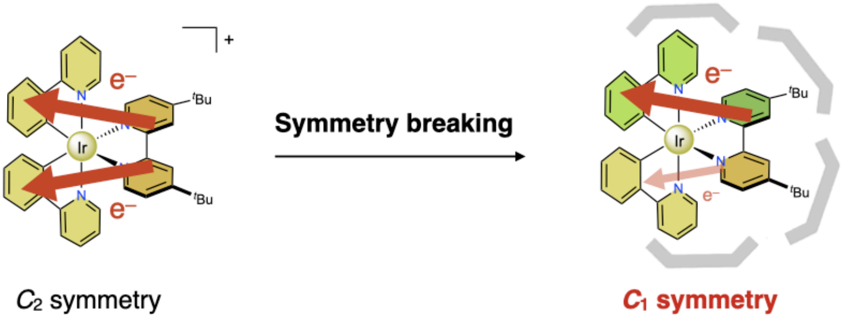

Supplementary Fig. 51 The vectors show de-excitation transitions (emission). The transition electronic dipole (ED) moment and the transition magnetic dipole (MD) moments were related to the vectors of the electron transition. As the free complex possesses C_2 symmetric structure, electron transition from bpy to ppy moieties is averaged. After vibrational relaxation of the excited state structure upon sensing the asymmetric environment originated from the symmetry-breaking assembly, the ppy ligands are located in different environments to give different contributions in the electron transition, hence enhancing the dissymmetry of the luminescence.

Supplementary References

1 Tunstad, L. M., Tucker, J. A., Dalcanale, E., Weiser, J., Bryant, J. A., Sherman, J. C., Helgeson, R. C., Knobler, C. B. & Cram, D. J. Host-guest complexation. 48. Octol building blocks for cavitands and carcerands. *J. Org. Chem.* **54**, 1305–1312 (1989).

2 Tian, N., Thiessen, A., Shiewek, R., Schmitz, O. J., Hertel, D., Meerholz, K. & Holder, E. Efficient Synthesis of Carbazolyl- and Thienyl-Substituted β-Diketonates and Properties of Their Red- and Green-Light-Emitting Ir(III) Complexes. *J. Org. Chem*. **74**, 2718–2725 (2009).

3 Helms, M., Lin, Z., Gong, L., Harms, K. & Meggers, E. Method for the Preparation of Nonracemic Bis-Cyclometalated Iridium(III) Complexes. *Eur. J. Inorg. Chem,* **2013**, 4164–4172 (2013).

4 Ito, A. & Matsui, Y. Electrochemical and Spectroscopic Behaviors of a Novel Ruthenium(II) Complex with a Six-Membered Chelate Structure. *Inorg. Chem.* **58**, 10436–10443 (2019).

5 Yamanaka, M., Shivanyuk, A. & Rebek, J. Jr. Kinetics and Thermodynamics of Hexameric Capsule Formation. *J. Am. Chem. Soc. 126*, 2939–2943 (2004).

6 Innocenzi, P., Kozuka, H. & Yoko, T. Fluorescence Properties of the $Ru(bpy)_3^{2+}$ Complex Incorporated in Sol-Gel-Derived Silica Coating Films. *J. Phys. Chem. B* **101**, 2285–2291 (1997).
